# Escape from NK cell tumor surveillance by NGFR-induced lipid remodeling in melanoma

**DOI:** 10.1126/sciadv.adc8825

**Published:** 2023-01-13

**Authors:** Julia Lehmann, Nicole Caduff, Ewelina Krzywińska, Salome Stierli, Adrian Salas-Bastos, Benjamin Loos, Mitchell P. Levesque, Reinhard Dummer, Christian Stockmann, Christian Münz, Johanna Diener, Lukas Sommer

**Affiliations:** ^1^ University of Zurich, Institute of Anatomy, Winterthurerstrasse 190, 8057 Zürich, Switzerland.; ^2^ University of Zurich, Institute of Experimental Immunology, Winterthurerstrasse 190, 8057 Zürich, Switzerland.; ^3^ University of Zurich Hospital, Department of Dermatology, Gloriastrasse 31, 8091 Zürich, Switzerland.

## Abstract

Metastatic disease is a major cause of death for patients with melanoma. Melanoma cells can become metastatic not only due to cell-intrinsic plasticity but also due to cancer-induced protumorigenic remodeling of the immune microenvironment. Here, we report that innate immune surveillance by natural killer (NK) cells is bypassed by human melanoma cells expressing the stem cell marker NGFR. Using in vitro and in vivo cytotoxic assays, we show that NGFR protects melanoma cells from NK cell–mediated killing and, furthermore, boosts metastasis formation in a mouse model with adoptively transferred human NK cells. Mechanistically, NGFR leads to down-regulation of NK cell activating ligands and simultaneous up-regulation of the fatty acid stearoyl–coenzyme A desaturase (SCD) in melanoma cells. Notably, pharmacological and small interfering RNA–mediated inhibition of SCD reverted NGFR-induced NK cell evasion in vitro and in vivo. Hence, NGFR orchestrates immune control antagonizing pathways to protect melanoma cells from NK cell clearance, which ultimately favors metastatic disease.

## Teaser

The neural crest stem cell marker NGFR drives melanoma cell metastasis by suppressing NK cell immunosurveillance.

## INTRODUCTION

Cutaneous melanoma is the deadliest form of skin cancer due to its high metastatic potential (*1*). An important hallmark of metastasis formation is tumor-instructed remodeling of the immune microenvironment toward an immune suppressive milieu enabling, first, tumor cell survival and maintenance and, second, dissemination, survival in the circulation, and homing to distant organs (*2*, *3*). Hence, commonly used immunotherapies, that mainly rely on boosting antitumor T cell response, such as PD-1/PD-L1 and CTLA4 checkpoint inhibitors, have brought great advances for patients with metastatic melanoma (*3*, *4*). Unfortunately, most of the patients still develop resistance to such checkpoint blockade, which highlights the need for alternative therapeutic strategies to manage metastatic melanoma (*5*).

More recently, innate immunity has gained attention for its crucial function in combatting metastatic disease (*6*). In particular, natural killer (NK) cells exert potent antitumor functions by releasing preformed cytotoxic granules containing granzyme and perforin, which rapidly trigger cytolysis of target cells. In contrast to T cells, NK cells are activated by an interplay of signals received by germ line–encoded activating and inhibitory receptors (*7*–*9*). Thus, their innate ability of recognizing transformed cells allows NK cells to kill without prior antigen sensitization and, importantly, to sense tumor cells with down-regulated major histocompatibility complex (MHC) class I—a commonly seen T cell evasion strategy in immunotherapy-resistant tumors (*10*, *11*).

While NK cell infiltration into solid tumors is often rather low (*8*), they are crucial for patrolling through vessels to eliminate circulating tumor cells (*12*). Depletion or inhibition of NK cells in immunocompetent mice increases metastasis (*13*, *14*), whereas NK cell adoptive transfer and activation of NK cells counteract metastatic dissemination (*15*, *16*). Specialized highly cytotoxic NK cells have been found in tumor-infiltrated lymph nodes (*17*) and metastases of patients with melanoma (*18*). Furthermore, proper NK cell function was found to be crucial for successful treatment of metastatic melanoma with commonly used targeted therapies such as BRAF inhibitors (*19*), suggesting NK cell–based therapies as combinatorial options for patients with melanoma (*8*, *12*).

The combined data provide cumulating evidence that NK cells are important guardians of tumor spreading. In the present study, we demonstrate how subpopulations of melanoma cells nevertheless are able to trick those key players of innate immunity. Specifically, we found that the overexpression of the nerve growth factor receptor NGFR (CD271/p75^NTR^) in melanoma cells led to reduced NK cell infiltration into melanoma xenografts, reduced NK cell–mediated tumor cell killing in vitro and in vivo, and increased metastasis formation in a mouse model with adoptively transferred human NK cells. Mechanistically, NGFR down-regulated activating NK cell ligands such as CD112 (Nectin-2) and CD155 (PVR). Furthermore, NGFR up-regulated sets of cholesterol and fatty acid metabolism genes, inducing changes in the lipid constitution of melanoma cells associated with strongly suppressed NK cell cytotoxicity toward NGFR^high^ melanoma cells. In particular, NGFR-driven expression of stearoyl–coenzyme A desaturase 1 (SCD), which catalyzes a rate-limiting step in the synthesis of unsaturated fatty acids, increased melanoma cell membrane fluidity and hampered the surface expression of NK cell activating ligands such as CD112. All in all, this interfered with NK cell cytotoxicity against melanoma cells and, consequently, inhibition of SCD efficiently restored impaired NK cell killing of NGFR^high^ tumor cells in vitro and in vivo. Because NGFR has been repeatedly linked to metastatic potential of melanoma (*20*–*23*) and to resistance development during immunotherapy (*24*–*26*), our findings on how NGFR promotes escape from the potent innate immune control of disseminated, metastatic cells may help designing future therapy approaches for metastatic melanoma.

## RESULTS

### NGFR regulates an innate immune evasion program

**Fig. 1.**
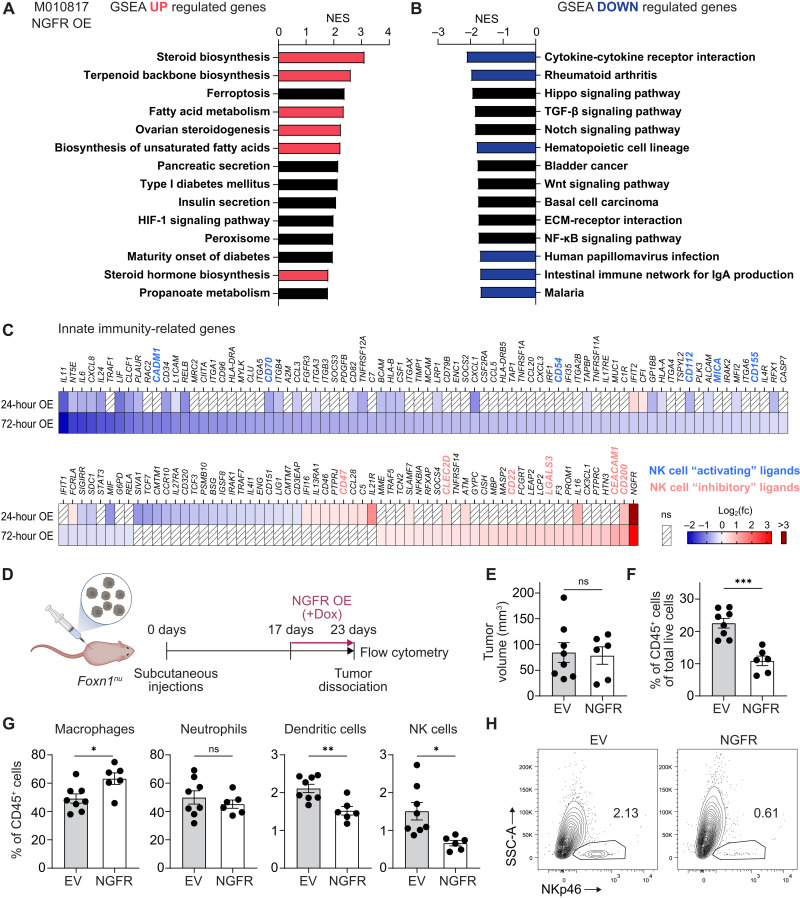
NGFR mediates an innate immune evasion signature. (**A** and **B**) GSEA of differentially expressed (DE) genes from RNA-seq of doxycycline (Dox)–inducible NGFR-overexpressing M010817 cells [comparing Dox-induced (24 hours) versus noninduced cells]. Genes were filtered for log_2_(fc) ≥ +0.27 or ≤ −0.27 and false discovery rate (FDR) < 0.05. Enrichment is based on the Kyoto Encyclopedia of Genes and Genomes (KEGG) pathway database showing top up-regulated (A) and top down-regulated pathways (B). Lipid biosynthesis–related pathways are highlighted in red, immune-related pathways in blue. NES, normalized enrichment score with *P* < 0.05. ECM, extracellular matrix. (**C**) Heatmap of DE innate immunity-related genes (InnateDB) after 24 and 72 hours of NGFR overexpression (Dox-induced versus noninduced cells). Genes encoding putative NK cell activating and inhibitory ligands are highlighted in blue and red, respectively. Shown are mean log_2_(fc) values of *n* = 3 with false discovery rate (FDR) < 0.05. OE, overexpression. (**D**) Illustration of experimental setup to investigate innate immune infiltrates. M010817 cells carrying Dox-inducible NGFR overexpression or empty vector (EV) control constructs were subcutaneously grafted into *Foxn1^nu^* mice. Seventeen days after engraftment (D17), mice were systemically treated with Dox in drinking water. Six days after onset of Dox treatment (D23), tumors were analyzed by flow cytometry. (**E**) Tumor volume. (**F**) Percentage of CD45^+^ cells of total live cells in tumors. (**G**) Percentage of macrophages, neutrophils, dendritic cells, and NK cells of tumor infiltrating CD45^+^ cells. (**H**) Representative flow cytometry blots of NKp46^+^ cells out of total CD45^+^ cells in EV and NGFR-induced tumors. Mean ± SEM. *P* values were calculated by unpaired, two-tailed Student’s *t* test with ∗*P* < 0.05, ∗∗*P* < 0.01, and ∗∗∗*P* < 0.001. ns, not significant.

Melanoma cell–intrinsic mechanisms of metastasis formation have been intensively studied (*27*–*29*). Among others, elevated expression of NGFR on melanoma cells has recently emerged as a key driver of invasiveness and metastasis formation (*20*, *21*, *23*). However, analysis of NGFR-regulated gene sets did not reveal any correlation with established invasiveness programs in melanoma cells (*27*–*29*), raising the question of how NGFR might promote metastasis formation. To address this issue, we first reanalyzed preexisting RNA sequencing (RNA-seq) data from melanoma cells overexpressing NGFR for 24 hours (data file S1) (*23*). In line with our previous study, gene set enrichment analysis (GSEA) of up-regulated genes after NGFR overexpression showed enrichments for cholesterol and fatty acid biosynthesis pathways (Fig. 1A and data file S2). Notably, dysregulation of lipid metabolism and cholesterol in cancer has been associated not only with cell invasion and metastasis (*30*) but also with suppression of the immune response against malignant cells (*31*–*33*). On the other hand, GSEA showed that NGFR overexpression led to down-regulation of genes related to immune response processes, such as “cytokine-cytokine receptor interaction” (Fig. 1B). Given the importance of innate immune cells in tumor surveillance and particularly the potent antimetastatic functions of NK cells (*34*), we specifically searched our dataset for changes in innate immune response genes [InnateDB (*35*)]. We found that NGFR induction led to decreased expression of several inflammatory cytokines like interleukin-11 (IL-11), IL-6, and CXCL8 (Fig. 1C and fig. S1A). This effect was even more pronounced after 72 hours of NGFR overexpression, revealing decreased expression of cytokines involved in innate immune cell recruitment [e.g., CXCL1, CXCL3, and CCL3 (*36*)], activation [e.g., MIF (*36*)], and differentiation [e.g., CSF-1 (*37*); Fig. 1C and fig. S1A]. Thus, NGFR-induced transcriptional changes within the tumor cells might be associated with evasion from recognition by innate immune cells.

To address this hypothesis, we overexpressed NGFR in human melanoma xenografts and analyzed tumor-infiltrating innate immune cells. Specifically, we subcutaneously injected patient-derived human melanoma cells engineered with a doxycycline (Dox)–inducible NGFR or empty vector (EV) control construct into athymic nude mice (Fig. 1D and fig. S1B). As those mice lack most of T cells but retain a fully functional innate immune system, they have been shown to exert a more pronounced innate immune response compared to euthymic mice (*38*, *39*), making them a suitable model to study innate immunity. Two and a half weeks after tumor cell engraftment, mice were systemically treated with Dox to induce NGFR expression. Six days later, mice were euthanized and tumors were analyzed for immune cell infiltrates by flow cytometry (fig. S1C). The four smallest NGFR-overexpressing tumors had to be excluded from the analysis (fig. S1D) because they did not provide enough tissue for downstream analysis, reflecting the previously described antiproliferative effect of NGFR (*20*, *21*, *23*). However, when analyzing the remaining tumors of comparable sizes (Fig. 1E), we discovered a notable reduction of CD45^+^ infiltrating immune cells in NGFR-overexpressing tumors compared to control tumors (Fig. 1F). The frequencies of all analyzed immune cell subtypes—macrophages, neutrophils, dendritic cells, and NK cells—were reduced upon NGFR overexpression (fig. S1E). Moreover, within the immune cell compartment (CD45^+^ cells), the composition changed toward more macrophages, less dendritic cells, and, importantly, also less NK cells (Fig. 1, G and H). Thus, these data suggest that NGFR elicits an innate immune evasive phenotype in melanoma.

### NGFR down-regulates NK cell ligands and interferes with NK cell activation

Intriguingly, within the immunosuppressive gene signature induced by NGFR, we found gene expression changes of several ligands important for regulating NK cell activity (Fig. 1C). Specifically, expression of ligands for activating NK cell receptors such as CADM1, CD70, CD54, CD112, MICA, and CD155 was decreased, while expression of ligands for inhibitory NK cell receptors such as CLEC2D, LAGLS3, CD22, CD47, CD200, and CEACAM1 was increased, suggesting an overall inhibitory effect on NK cell activity (*8*).

**Fig. 2.**
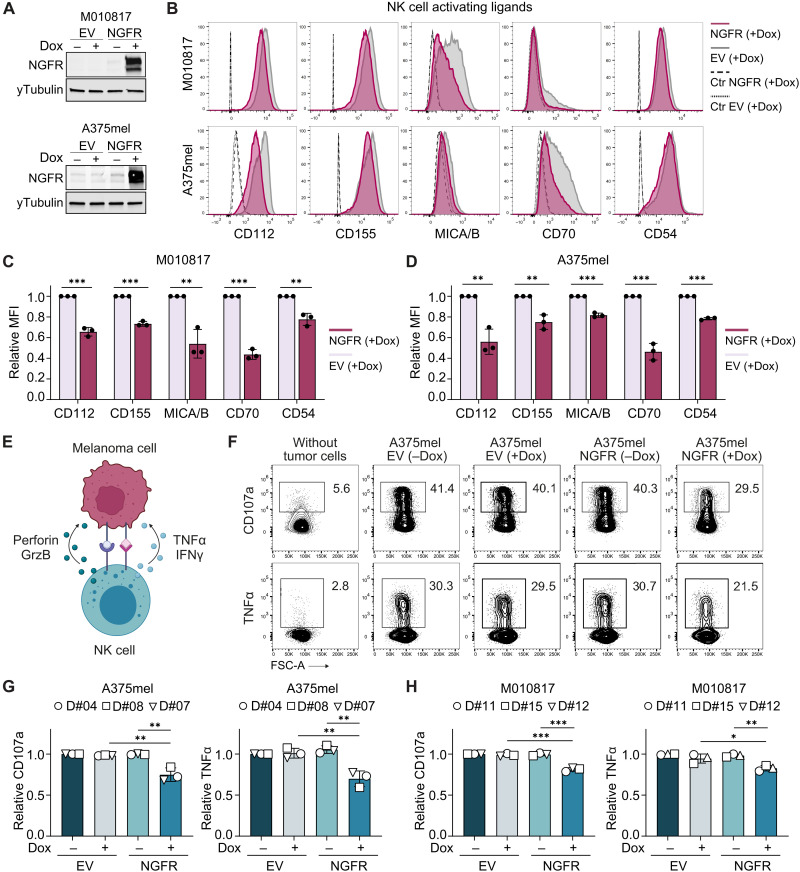
NGFR down-regulates ligands for activating NK cell receptors and lowers NK cell activation. (**A**) Western blot of EV and NGFR vector carrying M010817 and A375mel human melanoma cells after 72 hours of Dox treatment. (**B**) Representative flow cytometry histograms of NGFR (red) and EV (gray) melanoma cells 72 hours after Dox treatment and stained for ligands of activating NK cell receptors. Unstained cells are shown as controls (Ctr). (**C** and **D**) Mean fluorescent intensity (MFI) relative to EV control cells for M010817 (C) and A375mel cells (D). *N* = 3, means ± SD. (**E**) Illustration of NK cell activating and inhibitory receptors binding their corresponding ligands on the tumor cell leading to NK cell activation and release of cytotoxic granules containing perforin and granzyme B and release of cytotoxic cytokines TNFα and IFNγ. GrzB, granzyme B. (**F**) Representative flow cytometry plot of CD107a and intracellular TNFα on human NK cells (donor number 7; D#07) after 5-hour coculture with A375mel melanoma cells pretreated with Dox (for 72 hours). (**G** and **H**) Summary of CD107a and TNFα expression analysis by flow cytometry as in (F) based on human NK cells from three different donors (D#) after coculture with A375mel (G) and M010817 (H). Data shown relative to NK cells cocultured with EV control cells without Dox-treatment (−Dox). Circles, squares, and triangles represent mean values of technical duplicates or triplicates with cells from the same NK cell donor. *N* = 3, means ± SD. *P* values were calculated by unpaired, two-tailed Student’s *t* test with ∗*P* < 0.05, ∗∗*P* < 0.01, and ∗∗∗*P* < 0.001.

First, we validated whether differential expression of NK cell ligands indeed deregulates NK cell ligands at the protein level on the tumor cell surface. To do so, we performed flow cytometry–based cell surface analysis of well-established NK cell ligands on two human melanoma cell lines (M010817 and A375mel) carrying the Dox-inducible NGFR or EV control construct (Fig. 2A). Induction of NGFR lowered expression of the NK cell activating ligands CD112, CD155, MICA/B, CD70, and CD54 on melanoma cells (Fig. 2, B to D, and fig. S2A). Besides activating receptors, NK cells also carry NKG2A, an inhibitory receptor that senses as a heterodimer with CD94 the nonclassical human leukocyte antigen (HLA)–E. However, HLA-E was expressed at such low levels on our melanoma cells (fig. S2B) that its function can well be neglected. Moreover, NK cells recognize classical HLA class I molecules (HLA-A/HLA-B/HLA-C) through binding by inhibitory and activating killer immunoglobulin-like receptors (KIRs). Among the classical class I HLAs, HLA-C represents the predominant ligand for KIRs (*40*, *41*). However, mRNA expression of HLA-C was unaffected in melanoma cells upon NGFR induction (data file S1), suggesting no interference with NK cell activity.

Next, we evaluated whether NGFR-mediated ligand changes on tumor cells interfere with NK cell activation. For this purpose, we isolated human NK cells from the blood of healthy donors, cocultured them with human melanoma cells overexpressing NGFR, and measured NK cell activation by analyzing the degranulation marker CD107a and the intracellular synthesis of the cytokines tumor necrosis factor–α (TNFα) and interferon-γ (IFNγ) by flow cytometry (Fig. 2E). Intriguingly, human NK cells degranulated less and showed reduced synthesis of TNFα after cocultivation with NGFR-overexpressing melanoma cells compared to control cells (Fig. 2F). These findings were consistent for NK cells derived from different healthy donors (Fig. 2, G and H). IFNγ was generally detected only at very low levels, but its expression appeared to be reduced as well (fig. S2, C to F). Together, these data show that NGFR impairs NK cell activation by affecting both NK cell degranulation and cytokine production.

### NGFR expression protects human melanoma cells from NK cell lysis

**Fig. 3.**
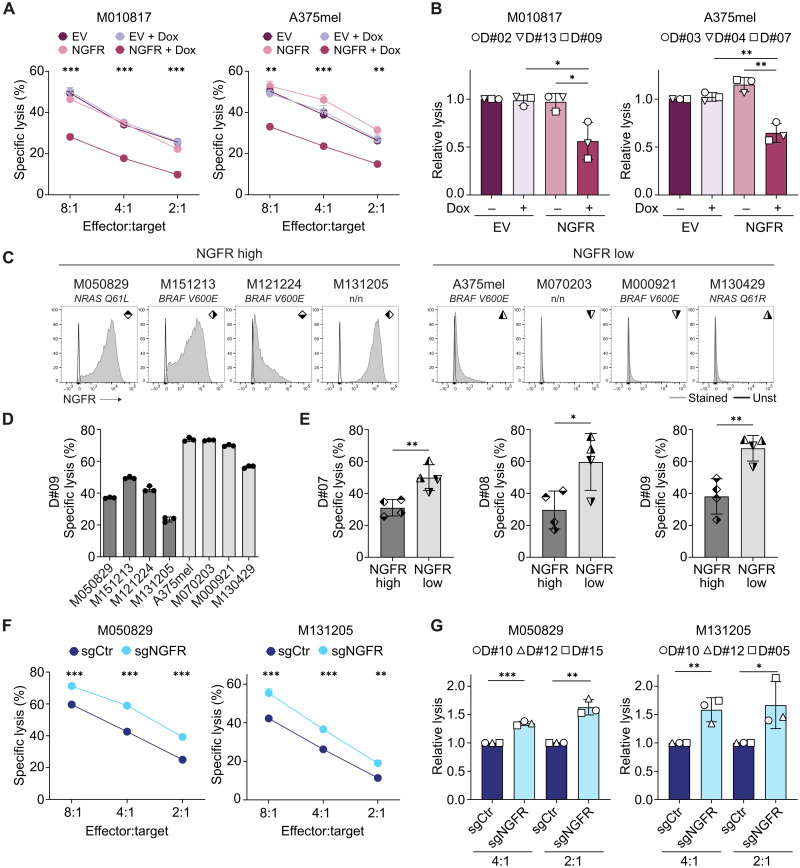
NGFR^high^ melanoma cells are less susceptible to NK cell–mediated killing. (**A**) Specific lysis measured by flow cytometry of EV or NGFR melanoma cells pretreated with (+Dox) or without Dox and cocultured with human NK cells (4 hours) at indicated effector to target cell ratios. Technical *n* = 2 to 4, means ± SD. (**B**) Relative lysis of melanoma cells normalized to EV (−Dox) control. Summary of independent experiments with NK cells from three different donors (D#) at 2:1 effector:target cell ratio. Circles, squares, and triangles represent means of technical duplicates or triplicates with cells from the same NK cell donor. *N* = 3, means ± SD. (**C**) Flow cytometry analysis of human melanoma cells with different mutational backgrounds (n/n, non-BRAF/non-NRAS mutant). (**D**) Specific lysis of cells from (C) after 4-hour coculture with human NK cells (D#09) at 4:1 effector: target cell ratio. Technical *n* = 3, means ± SD. (**E**) Specific lysis summary of cells from (C) with NK cells from three different donors (D#07, D#08, and D#09). Squares and triangles represent means of technical duplicates or triplicates from the respective cell line. Means ± SD. (**F**) Specific lysis of melanoma cells 6 days after CRISPR-induced gene knockout with a Dox-inducible single control guide (sgCtr) or single guide targeting NGFR (sgNGFR). Technical *n* = 3, means ± SD. (**G**) Relative lysis of melanoma cells normalized to sgCtr. Circles, squares, and triangles represent means of technical duplicates or triplicates with cells from the same NK cell donor (D#). *N* = 3, means ± SD. *P* values were calculated by unpaired, two-tailed Student’s *t* test with ∗*P* < 0.05, ∗∗*P* < 0.01, and ∗∗∗*P* < 0.001.

To address whether NGFR-induced suppression of NK cell activation results in tumor cell resistance toward NK cell attack, we cocultured human NK cells together with melanoma cells and measured their lysis by a flow cytometry–based approach. Tumor cells overexpressing NGFR were less killed by NK cells compared to control cells, an effect that was found at different effector to target cell ratios and consistent over different NK cell donors (Fig. 3, A and B). To ensure that this immunosuppressive phenotype was not an artifact of the genetic engineering of NGFR-inducible cells but due to NGFR itself, we further carried out experiments with human melanoma cells endogenously expressing high levels of NGFR. Supporting the specific role of NGFR in NK cell suppression, we found that intrinsically NGFR^high^ melanoma cells were less susceptible to NK cell killing than NGFR^low^ melanoma cells (Fig. 3, C to E). Last, we consolidated our findings by knocking out NGFR in the NGFR^high^ cells M050829 (fig. S2, G and H) and M131205 (fig. S2H), which led to increased cytolysis by NK cells (Fig. 3, F and G). Together, these findings show that NGFR renders melanoma cells less susceptible to NK cell–mediated lysis.

### NGFR expression also curbs responses of KIR-matched NK cells

**Fig. 4.**
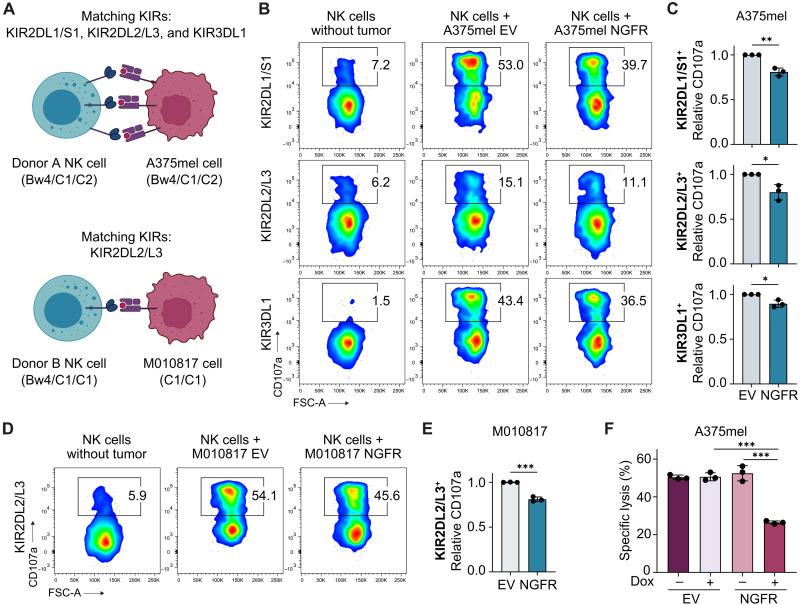
Degranulation and cytotoxicity of KIR-matched NK cells are impaired upon NGFR expression. (**A**) Schematic illustration of KIR molecules on NK cells binding cognate HLA molecules on tumor cells. Top: Donor A–derived NK cells and A375mel cells match in KIR2DL1/S1, KIR2DL2L3, and KIR3DL1. Bottom: Donor B–derived NK cells and M010817 cells match in KIR2DL2/L3. HLA-B/HLA-C types are shown in brackets. (**B**) Flow cytometry analysis of CD107a on indicated KIR-positive NK cells from donor A after 5-hour coculture with matching A375mel cells (EV or NGFR) pretreated with Dox for 72 hours. (**C**) Quantification of (B) showing mean values of three independent experiments, each performed in technical duplicates or triplicates. Means ± SD. (**D** and **E**) Same as (B) and (C) for donor B–derived NK cells and M010817 target cells. Means ± SD. (**F**) Specific lysis of A375mel tumor cells after coculture with KIR-matched donor A–derived NK cells at a 4:1 effector:target ratio. Data are representative of three independent experiments. Means ± SD. *P* values were calculated by unpaired, two-tailed Student’s *t* test with ∗*P* < 0.05, ∗∗*P* < 0.01, and ∗∗∗*P* < 0.001.

Using NK cells from randomly selected healthy donors, we found that overexpression of NGFR in melanoma cells led to a reduced NK cell response. NK cells get educated via the interaction between KIR molecules and their cognate self-MHC class I ligands and become increasingly responsive to MHC-deficient target cells and more receptive to activating stimuli (*42*, *43*). Therefore, an HLA class I mismatch between NK and target cells derived from different donors may bias the outcome of NK cell responses. To make sure that this was not the case, we repeated our experiments in a KIR-matched setting: We HLA-haplotyped M010817 and A375mel cells and specifically assessed the degranulation of matched KIR^+^ NK cells (Fig. 4A and fig. S3A). We found that KIR (KIR2DL1/S1, KIR2DL2/L3, and KIR3DL1)–positive NK cells from donor A (HLA-Bw4/C1/C2), educated by cognate HLA molecules as expressed by A375mel cells (HLA-Bw4/C1/C2), degranulated less when cocultured with NGFR-overexpressing A375mel cells compared to EV control cells (Fig. 4B and fig. S3B), as summarized for three independent experiments in Fig. 4C. Likewise, KIR (KIR2DL2/L3)–positive NK cells from donor B (HLA-Bw4/C1/C1), educated by cognate HLA molecules as expressed by M010817 cells (HLA-C1/C1), degranulated less when cocultured with NGFR-overexpressing M010817 cells compared to EV control cells (Fig. 4, D and E, and fig. S3C). Notably, NGFR-overexpressing and control tumor cells activated similar NK cell subsets, as shown by Uniform Manifold Approximation and Projection (UMAP) visualization (fig. S3, D and E), indicating that tumor cell NGFR does not change the composition of NK cell subpopulations but rather imposes a general suppression on NK cell activity. Last, we found that NGFR-overexpressing cells in partial and full KIR-matched settings lead to a significant reduction in NK cell–directed tumor cell killing (Fig. 4F and fig. S3F). Together, we conclude that our previous observations were not due to alloreactivity between NK and tumor cells and that also educated KIR-positive NK cells are attenuated by autologous NGFR^high^ melanoma cells.

### NGFR protects melanoma cells from NK cell–directed lysis in vivo

**Fig. 5.**
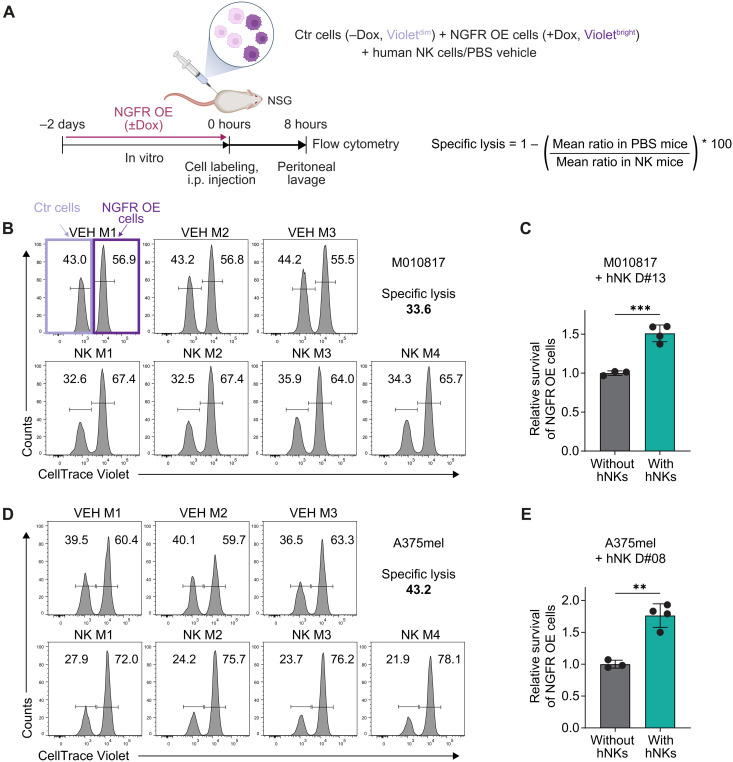
NGFR expression protects melanoma cells from human NK cell cytotoxicity in vivo. (**A**) Scheme depicting the experimental setup of the in vivo cytotoxic assay. Human melanoma cells carrying the NGFR-OE vector were pretreated with Dox in vitro for 48 hours to induce NGFR (NGFR OE) and labeled with a higher concentration (1 μM) of CellTrace Violet than non-Dox–treated control cells (0.1 μM). NGFR OE (Violet^bright^) and control (Violet^dim^) cells were mixed at equal numbers and injected together with human NK cells (at a 1:1 effector:target cell ratio) or PBS as vehicle control into the peritoneum of NSG mice. Eight hours after injection, cells were isolated by peritoneal lavage and analyzed by flow cytometry. Right: Formula to calculate specific lysis difference. i.p., intraperitoneal. (**B**) Flow cytometry analysis of CellTrace Violet–labeled M010817 cells recovered from individual mice (VEH M1-M3: mice injected with vehicle; NK M1-M4: mice injected with human NK cells). (**C**) Relative survival of NGFR OE cells (Violet^bright^) over control cells (Violet^dim^) in mice with human NK cells (hNKs) normalized to mice without human NK cells. Means ± SD. (**D** and **E**) Same as in (B) and (C) for A375mel cells. Means ± SD. *P* values were calculated by unpaired, two-tailed Student’s *t* test with ∗*P* < 0.05, ∗∗*P* < 0.01, and ∗∗∗*P* < 0.001.

Next, we asked whether NGFR also protects melanoma cells from NK cell killing in vivo. We first overexpressed NGFR in the human melanoma cells in vitro (Fig. 5A) and labeled these with different concentrations of a violet cell tracing dye to allow the discrimination of NGFR-overexpressing (Violet^bright^) and control (Violet^dim^) tumor cells in vivo. Equal numbers of NGFR-overexpressing and control cells were then mixed and injected together with human NK cells or vehicle [phosphate-buffered saline (PBS)] into the peritoneum of immunodeficient NOD-*scid* IL2Rg^null^ (NSG) mice. Eight hours after injection, cells were isolated by peritoneal lavage and analyzed by flow cytometry. Intriguingly, the relative frequency of NGFR-overexpressing cells increased in mice engrafted with human NK cells compared to mice without human NK cells, indicating preferential killing of NGFR^low^ control cells (Fig. 5, B to E). Thus, we conclude that NGFR confers a survival advantage to tumor cells in the presence of human NK cells in vivo.

### NK cell cytotoxicity is impaired by NGFR-mediated lipid remodeling in melanoma cells

**Fig. 6.**
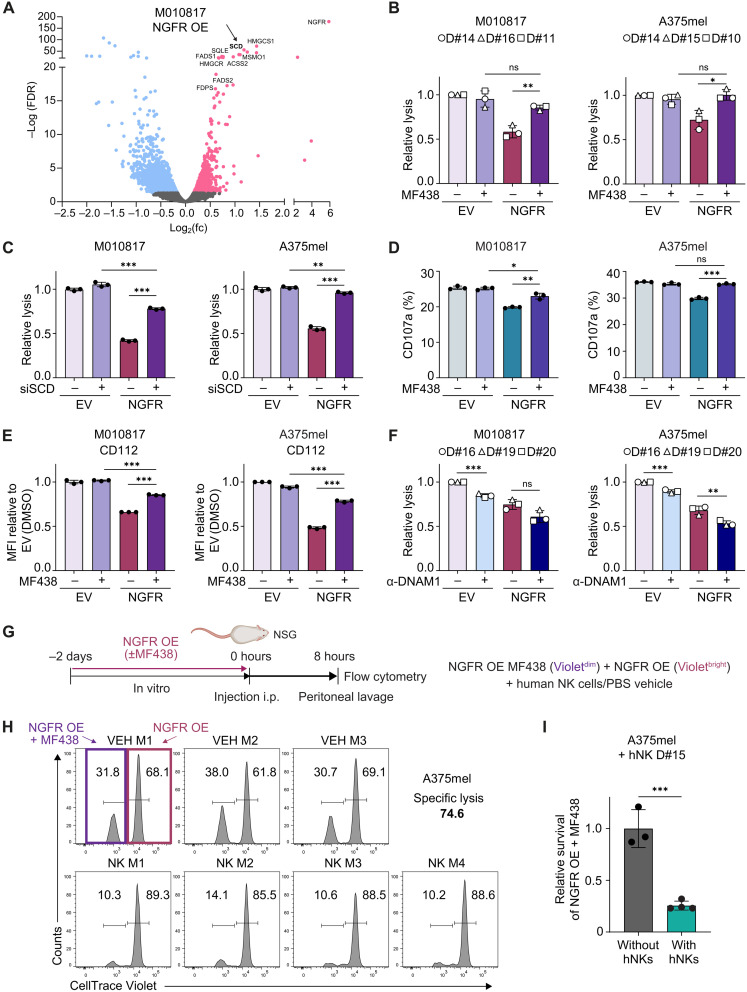
NGFR mediates evasion from NK cells by altering melanoma cell lipid metabolism. (**A**) Volcano plot of DE genes upon NGFR OE (as data in Fig. 1, A and B). (**B**) Relative lysis of melanoma cells pretreated with Dox and 10 μM MF438 or vehicle [dimethyl sulfoxide (DMSO)] for 48 hours from three independent experiments (technical *n* = 3) with different donors at 4:1 effector:target cell ratio. Means ± SD. (**C**) Relative lysis of melanoma cells pretreated with siCtr (−) or siSCD (+) for 72 hours and Dox for 48 hours at 4:1 effector:target cell ratio. Technical *n* = 3, means ± SD. (**D**) Flow cytometry analysis of CD107a on NK cells after coculture with melanoma cells. Technical *n* = 3, means ± SD. (**E**) Quantification of CD112 on melanoma cells pretreated with MF438 or vehicle (DMSO) and Dox for 48 hours. Technical *n* = 3, means ± SD. (**F**) Relative lysis of melanoma cells pretreated with Dox for 48 hours after coculture with human NK cells and anti–DNAM-1 antibody (10 μg/ml) for 4 hours. Means of three independent experiments (technical *n* = 3) with different NK cell donors at 4:1 effector:target cell ratio. Means ± SD. (**G**) Illustration of in vivo cytotoxic assay with MF438 or vehicle (DMSO) and Dox for 48-hour pretreated NGFR OE cells. (**H**) Flow cytometry analysis of CellTrace Violet–labeled cells recovered from mice (M#) injected with PBS vehicle (VEH) or human NK cells (NK). (**I**) Summary of (H) showing relative survival of NGFR OE cells + MF438 over NGFR OE cells + DMSO in mice with human NK cells normalized to mice without human NK cells. Means ± SD. *P* values were calculated by unpaired, two-tailed Student’s *t* test with ∗*P* < 0.05, ∗∗*P* < 0.01, and ∗∗∗*P* < 0.001.

So far, we had observed reduced NK cell activation and reduced NK cell–mediated cytolysis of NGFR^high^ melanoma cells in vitro and in vivo. Moreover, RNA-seq data had suggested that this was due to NGFR-mediated down-regulation of NK cell activating ligands in melanoma cells. However, because lipid remodeling—a pathway predominantly up-regulated by NGFR-overexpressing cells (Fig. 1A)—has also been associated with NK cell tumor immune evasion, we wondered whether this might represent an additional mode of action by NGFR to bypass NK cell surveillance. Within pathways enriched upon NGFR overexpression, genes like HMGCR, a key regulator of cholesterol synthesis, or SCD, the rate-limiting enzyme in fatty acid desaturation, were most significantly up-regulated (Fig. 6A). Strengthening the transcriptome results, untargeted lipidomic analysis revealed enrichment of metabolites belonging to sterol/cholesterol lipids, unsaturated fatty acids, and unsaturated glycerophospholipids in NGFR-overexpressing cells (fig. S4, A and B, and data file S3).

To examine whether lipid alterations play a role in NGFR-mediated attenuation of NK cells, we inhibited these lipid biosynthetic pathways in NGFR-overexpressing cells and assessed whether NK cell killing was impaired. Inhibition of cholesterol synthesis by lovastatin did not influence NGFR-induced reduction in NK cell lysis (fig. S4C). In contrast, impaired NK cell killing of NGFR-overexpressing cells could almost completely be rescued by treating NGFR^high^ melanoma cells with MF438, an inhibitor of the fatty acid desaturase SCD (Fig. 6B). Consistently, blocking of SCD in M050829 with high endogenous NGFR expression also enhanced susceptibility to NK cell lysis (fig. S4D). Moreover, small interfering RNA (siRNA)–mediated silencing of SCD almost completely restored impaired NK cell killing of NGFR-overexpressing cells, confirming an SCD-specific effect (Fig. 6C and fig. S4E). Besides killing efficacy, also NK cell activity was restored upon inhibiting SCD in NGFR-overexpressing cells (Fig. 6D and fig. S4, F and G). Notably, NGFR expression itself was not affected by SCD inhibition (fig. S4H).

Because we had found that NGFR overexpressing cells deregulate lipid metabolites (fig. S4, A and B) and because RNA-seq had additionally shown that NGFR down-regulates inflammatory cytokines (Fig. 1C)—both molecule classes known to be secreted and able to act as long-range, soluble cues—we next asked whether secreted factors from NGFR^high^ cells might impede NK cell cytotoxicity. However, conditioned media from NGFR^high^ tumor cells did not influence NK cell killing of parental (NGFR^low^) tumor cells (fig. S5A), suggesting that NGFR-mediated evasion from NK cell cytotoxicity does not primarily happen through secretory pathways but rather involves direct effector cell–target cell interactions.

Notably, we found that SCD inhibition led to the up-regulation of the NK cell ligands CD112 and CD155 on the surface of NGFR-overexpressing tumor cells (Fig. 6E and fig. S5, B and C) without significantly altering expression levels of other assessed NK cell activating ligands (fig. S5D). CD112 and CD155 can both signal through inhibitory receptors [TIGIT; (*44*, *45*)] or activating receptors [DNAM-1; (*46*, *47*)] on NK cells. Because in our system CD112/CD155 down-regulation correlated with reduced NK cell activation and reduced tumor cell killing, we next assessed whether signal activation through DNAM-1 is a key mediator for NK cell cytotoxicity against melanoma cells. To do so, we blocked DNAM-1 signaling using a neutralizing antibody and indeed found NK cell lysis of A375mel and M010817 melanoma cells to be significantly reduced (Fig. 6F). Upon combining NGFR overexpression with DNAM-1 blockade, NK cell cytotoxicity was further hampered. This was to be expected, because overexpression of NGFR resulted in down-regulation but not complete depletion of immune cell ligands (Fig. 2, B to D), suggesting that signaling through DNAM-1 is still active in presence of NGFR^high^ cells, albeit to a lower degree. However, the effect of DNAM-1 signaling blockade on NK cell antitumor lysis was not as strong as that of NGFR overexpression alone, suggesting that additional mechanisms of NK cell attenuation are involved in NGFR^high^ melanoma cell signaling (Fig. 6F).

A recent study suggests that membrane lipid composition of tumor cells likely influences successful docking and immunological synapse formation by NK cells (*48*). Given the important role of SCD in regulating phospholipid desaturation, which is known to affect cell membrane fluidity (*49*, *50*), we checked whether NGFR may alter cell membrane properties. Using fluorescent Laurdan dye, whose emission spectra are dependent on lipid packing density (*51*), we quantified the generalized polarization (GP) of melanoma cell plasma membranes (fig. S6, A and B). Intriguingly, cells overexpressing NGFR showed reduced GP, indicating a higher fluidity of their plasma membranes, which is consistent with higher levels of unsaturated phospholipids and higher SCD activity (fig. S6C). SCD inhibition reverted this phenotype, suggesting that NGFR mediates evasion from NK cell killing at least partially by changing membrane fluidity.

By blocking SCD, we were able to restore NK cell killing of NGFR-overexpressing melanoma cells in vivo (Fig. 6G), as reflected by a notable loss of MF438-treated NGFR-overexpressing cells in mice engrafted with human NK cells (Fig. 6, H and I). Conversely, survival of MF438-treated EV control cells compared to nontreated EV cells was only slightly affected by human NK cells in vivo (fig. S6, D and E). Thus, these data strongly support the notion that SCD-induced lipid remodeling is a specific mechanism regulated by NGFR to protect tumor cells from NK cell cytotoxicity.

### NGFR increases metastasis formation by mediating evasion from NK cell surveillance

**Fig. 7.**
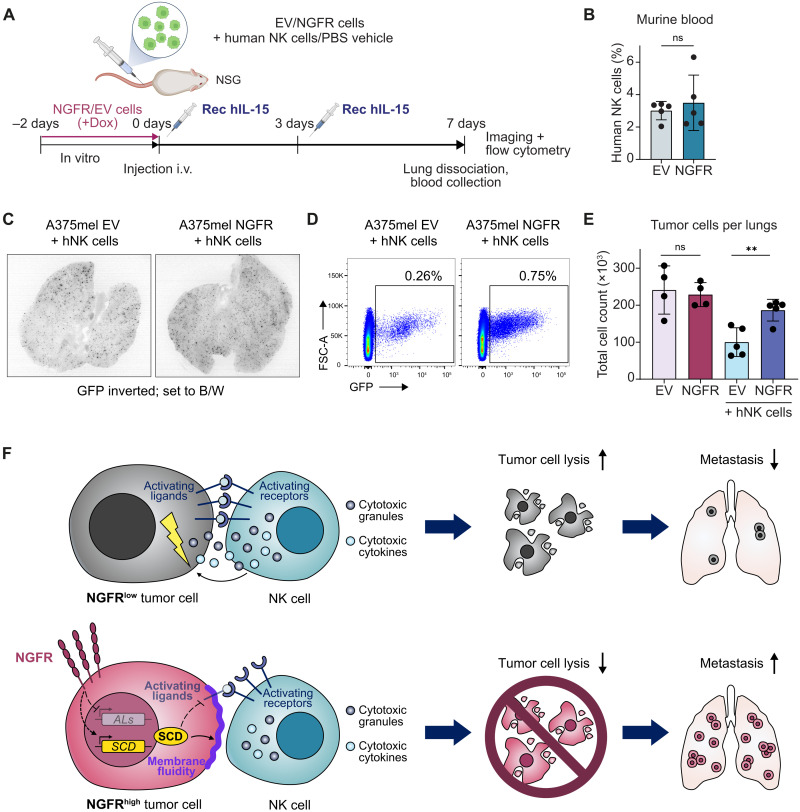
NGFR increases metastasis by mediating evasion from NK cell-directed lysis. (**A**) Illustration of experimental metastasis assay in NSG mice with adoptively transferred human NK cells. A375mel EV and NGFR cells pretreated with Dox for 48 hours were injected into tail veins of NSG mice in combination with human NK cells or PBS (vehicle control). At the day of cell injection (0 days) and 3 days later (3 days), recombinant human IL-15 (hIL-15) was intraperitoneally injected to sustain human NK cells. One week after cell grafting (7 days), lungs and blood were collected and analyzed for NK and tumor cells. i.v., intravenous. (**B**) Flow cytometry of human NK cells (human CD45^+^) in the murine blood (shown as percentage of whole-blood samples). Means ± SD. (**C**) Fluorescent binocular images of murine lungs with NGFR- and EV-inducible melanoma cells stably expressing GFP. Signal inverted and set to black/white (B/W). Images were acquired with different intensities and only serve as illustration. (**D**) Representative flow cytometry analyses of dissociated murine lung tissue with GFP^+^ EV or GFP^+^ NGFR tumor cells as depicted in (A). (**E**) Summary of flow cytometry analysis for total numbers of GFP^+^ melanoma cells in the lungs from mice with (*N* = 5) and without (*N* = 4) adoptively transferred human NK cells. Absolute numbers were quantified by normalizing to counting beads. Means ± SD. *P* values were calculated by unpaired, two-tailed Student’s *t* test with ∗*P* < 0.05, ∗∗*P* < 0.01, and ∗∗∗*P* < 0.001. (**F**) Model summarizing the data presented in this study (for details, see the main text). ALs, activating ligands.

Last, as NK cells are important guardians of circulating tumor cells, we asked whether NGFR-mediated NK cell evasion drives melanoma metastasis formation. To address this in a humanized system, we adoptively transferred human NK cells together with preinduced NGFR or EV control A375mel cells into NSG mice by tail vein injection (Fig. 7A). In addition, we intraperitoneally administered recombinant human IL-15 to maintain human NK cell survival. Seven days after adoptive transfer, we first confirmed similar frequencies of human NK cells in the blood of these two treatment groups (Fig. 7B). Next, we collected the lungs to check for tumor burden. Using fluorescent imaging, we observed that NGFR-induced melanoma cells had formed more metastases compared to EV control cells (Fig. 7C). Quantification of the total numbers of green fluorescent protein (GFP)–positive tumor cells in single-cell suspensions of the lungs by flow cytometry confirmed increased numbers of NGFR-induced cells compared to EV control cells in mice with transferred human NK cells (Fig. 7, D and E). Notably, in human NK cell–deficient mice, tumor cell numbers of NGFR-induced and control cells did not differ, indicating that evasion from NK cells is a key process in NGFR-mediated melanoma metastasis formation. In sum, we found that NGFR confers innate immune escape to melanoma cells by regulating immune control antagonizing pathways, namely, the suppression of NK cell activating ligands and the activation of fatty acid desaturation. Together, this leads to reduced killing of tumor cells by NK cells and, eventually, to increased metastasis formation (Fig. 7F).

## DISCUSSION

Melanoma is a highly immunogenic tumor, and therefore, multiple approaches to boost antimelanoma immunity have been explored (*52*). However, it has become evident that tumors exploit an array of strategies to trick the immune system. Melanoma is a very heterogeneous tumor (*53*), and emergence of subpopulations resistant to immune control or immunotherapy is a major challenge in patient treatment (*54*, *55*). Therefore, understanding the various melanoma phenotypes leading to immune evasion is crucial for specific treatments of different patient disease courses.

It has been repeatedly found that subpopulations within melanoma express the neural crest stem cell marker NGFR, which has been linked to tumor initiation, stress responses, phenotype switching, metastasis formation, and therapy resistance (*20*, *21*, *23*, *24*, *56*, *57*). In addition, NGFR has also been shown to be functionally involved in providing resistance to melanoma-specific T cell attack in vitro (*26*). Complementary to previous studies claiming an inverse correlation between NGFR expression and melanoma-infiltrating CD8^+^ T cells (*24*–*26*), we report here reduced infiltration of innate immune cells in NGFR^high^ melanoma xenografts, suggesting that NGFR regulates a general immunosuppressive program in melanoma. Specifically, we show that NGFR helps melanoma cells to evade innate immune surveillance by human NK cells, leading to metastasis formation. Our conclusions are not biased because of artifacts of alloreactivity between cells from different human donors, because NGFR also confers protection from NK cell lysis to KIR-matched tumor cells.

Further evidence supporting a link between NGFR and innate immunity comes from a recent melanoma study wherein tumors deficient in phospho-eIF4E, a translation initiation factor regulating, among others, NGFR, showed increased infiltration of tumor-promoting myeloid-derived suppressor cells (*58*). Moreover, metastasis samples from a patient with relapsing disease had, in proximity to immune cells, tumor cells displaying up-regulated NGFR and, at the same time, the immune checkpoint ligand PD-L1 (*59*).

Another study reported that after challenging melanoma cells with NK cells, the tumor cells underwent an EMT-like phenotype switch from proliferative to invasive (*60*). In line with this, previous studies reported up-regulation of NGFR upon challenge with TNFα and IFNγ (*24*, *61*)—the main cytokines released upon activation of NK cells and T cells. Vice versa, it has been shown that melanoma cells with high dissemination capacity express NGFR, which has further been validated as a marker for circulating melanoma cells (*62*). In a previous study we demonstrated that, when temporally overexpressed, NGFR is a potent inducer of melanoma phenotype switching (*23*). This is again in accordance with the general idea that stem cell–like cancer cell states in many tumors are linked to immune evasion (*63*) and that immune evasion is linked to phenotype switching in melanoma (*58*, *60*, *64*).

While phenotype switching (EMT) and stem cell–like melanoma cells have been associated with NK cell evasion (*60*, *65*), other studies have found that melanoma cells expressing MITF, the master regulator of melanocytic differentiation (*66*), can also escape NK cell lysis (*67*, *68*). Mechanistically, this has been linked to MITF-induced up-regulation of the metallopeptidase ADAM10, which can cleave the MICA/B family of NK cell ligands (*67*). However, in our study, ADAM10 expression was not altered upon NGFR overexpression, suggesting another molecular mechanism of NK cell evasion. Also, the relationship between MITF and NGFR remains unclear: While NGFR expression is elevated in melanoma with low MITF and has been identified as a direct target of MITF (*69*), we have not observed a consistent effect of NGFR overexpression on MITF expression in tumors in vivo (*23*). Likewise, while many stress responses in melanoma cells were associated with high NGFR and low MITF expression, other stress and invasiveness-inducing conditions resulted in increased NGFR levels but without affecting MITF expression (*57*). All in all, we therefore propose that NK cell evasion by NGFR up-regulation is independent of MITF.

In our previous study, we associated the observed phenotype switch mediated by NGFR with cell-intrinsic changes in cellular adhesion (*23*). In particular, inhibition of NGFR-induced cholesterol and lipid biosynthesis could rescue NGFR-mediated loss of adhesion in vitro. While adhesion changes are key to primary tumor cells undergoing the first steps toward an invasive phenotype, we now show in this study that NGFR-mediated up-regulation of the fatty acid desaturase SCD is a central step in melanoma innate immune evasion from NK cells and, consequently, increased metastasis formation. This is in line with numerous other studies associating increased SCD activity with cancer cell aggressiveness [reviewed in (*70*)]. In contrast, others identified SCD as a suppressor of inflammatory signaling that inhibited metastasis formation (*68*), revealing potentially distinct effects of SCD in tumorigenesis.

We propose that one mechanism by which SCD mediates melanoma cell protection from NK cell killing is by reducing surface expression of CD112 and CD155. These ligands can activate NK cells upon binding to their DNAM-1 receptor (*47*, *71*), but they were more recently reported to also interact with the inhibitory receptors CD112R, TIGIT, or CD96 on NK cells (*72*–*74*). Here, we demonstrate an activating role of CD112 and CD155 that promote NK cell antimelanoma cytotoxicity by interacting with DNAM-1. However, as blocking of DNAM-1 signaling was not sufficient to lower NK cell antitumor lysis to a similar level as NGFR overexpression alone, we propose that other mechanisms, including in particular altered lipid metabolism, are involved.

Our study provides evidence that NGFR alters cell membrane fluidity by increasing the level of unsaturated phospholipids in an SCD-dependent manner. A recent study reported that changes in membrane lipid packing protected from perforin- and granzyme-mediated lysis (*48*). This supports the idea that NGFR^high^ cells reduce their susceptibility to NK cell lysis by altering their membrane composition, which could affect successful docking and immunologic synapse formation by NK cells. In addition, changes in membrane composition could affect cell surface expression of NK cell activating ligands, such as CD112 and CD155 (*75*).

As we have found a notable role of lipid metabolism on attenuating NK cell function, it is plausible that aberrant fatty acid metabolism is a key molecular pathway in NGFR-induced NK cell evasion by inducing fundamental tumor cell remodeling. This hypothesis is supported by emerging research correlating elevated tumor cell cholesterol and fatty acid metabolism with suppression of the anticancer innate immune response (*31*, *32*)—for instance, by regulating immunosuppressive tumor-associated macrophages (*76*) or by impairing NK cell effector functions (*33*, *77*, *78*). However, while these studies have mostly focused on the impact of lipids from dietary fats, we show in the present study that NGFR induces a cancer cell–intrinsic switch toward increased lipid metabolism. Intriguingly, the NGFR-mediated changes in lipid metabolism significantly impair antimelanoma cytotoxicity of NK cells, enabling increased cancer cell survival in the circulation and metastatic homing to secondary organs.

In conclusion, our findings suggest NGFR as promising therapeutic target in melanoma. By directly targeting this surface receptor, tumor cells that are highly invasive, self-sustaining, and immune evasive could be tackled. Humanized cytotoxic antibodies against NGFR are in preclinical trials (*79*) and might advance specific targeting of this aggressive melanoma cell subtype. Alternatively, to specifically avoid NK cell attenuation and eliminate NGFR^high^ cells—which have additionally been shown to emerge as resistant cells toward common melanoma therapies (*56*)—genetically engineered CAR-NGFR NK cells with cytotoxicity enhancers could be used in combination with conventional melanoma therapies. NK cells are known for their low off-target toxicity and high intrinsic natural cytotoxicity against tumor cells, making them excellent candidates for immunotherapy, which is supported by multiple clinical trials (*8*). NK cell–based therapies are of particular interest when targeting tumor cells that lack suitable surface antigens and hence escape from T cell clearance. Moreover, NK cells—isolated from peripheral blood, cord blood, or induced pluripotent stem cells—are also discussed as “off-the-shelf” therapeutic product because they can be used in allogeneic settings and do not cause graft-versus-host-disease (*8*). Last, SCD inhibitors are also in clinical trials (*70*), and interfering with the aberrant lipid metabolism in NGFR^high^ cells might be an alternative approach to directly targeting NGFR.

## MATERIALS AND METHODS

### Animal experimental models

Nude (Hsd:Athymic Nude-Foxn1^nu^) mice were purchased from Envigo. NSG (NOD.Cg-Prkdc^scid^ Il2rg^tm1Wjl^/SzJ) mice were purchased from Charles River or from The Jackson Laboratory and bred and maintained at the Institute of Experimental Immunology, University of Zurich. For short-term in vivo cytotoxic assays, 8- to 18-week-old NSG mice of both sexes were used. For long-term subcutaneous xenograft experiments and for experimental metastasis experiments, female mice of 16 weeks of age and female mice of 7 weeks of age, respectively, were used. Littermates of the same sex were randomly allocated to experimental groups. For experimental metastasis assays, tail vein injections were performed in a blinded manner. Animals were housed in a certified animal facility with a 12-hour light/dark cycle, with free access to water and food and at temperatures of 21° to 23°C and humidity of 40 to 60%. All animal experiments were approved by the veterinary office of Canton of Zurich, Switzerland and were performed in accordance with Swiss law.

### Human blood samples

Blood samples were either obtained from anonymized healthy adult donors provided by the Zurich blood donation services (BASEC-NR: Req-2020-00883) or from healthy adult volunteers upon informed consent. All experiments involving samples from human donors were conducted with the approval of the ethics committee of Canton of Zurich, Switzerland.

### Cell lines and primary cell cultures

The human cell lines A375mel [American Type Culture Collection (ATCC)] and human embryonic kidney (HEK)–293T (ATCC) were commercially purchased. Primary human melanoma short-term cell cultures M000921, M010817, M050829, M070203, M121224, M130429, M131205, and M151213 were established and provided by the University Priority Research Program (URPP) biobank at the University Hospital Zurich, Department of Dermatology. Primary melanoma cell cultures were generated from excess tumor material of surgically removed melanoma metastases from patients after written informed consent and approved by the local institutional review board (EK647 and EK800). Clinical diagnosis of tumor material was confirmed by histology and immunohistochemistry. HEK-293T cells were grown in Dulbecco’s modified Eagle’s medium (DMEM) supplemented with 10% heat-inactivated fetal bovine serum (FBS). Human melanoma cells were cultured in RPMI 1640 supplemented with 10% heat-inactivated FBS, 4 mM l-glutamine, and penicillin-streptomycin (complete RPMI) in a humidified incubator at 37°C and 5% CO_2_.

### NGFR overexpression in human melanoma cells

Human melanoma cells stably transduced with the Dox-inducible CMVTOEV and CMVTONGFR constructs (*23*) were treated with Dox (1 μg/ml; Sigma-Aldrich) in complete RPMI 1640 medium to induce overexpression. Time points of overexpression are indicated for individual experiments, with 24-hour overexpression referring to 24-hour Dox treatment and 72-hour overexpression referring to 24-hour Dox treatment followed by 48-hour culture without Dox.

### RNA-seq data analysis

The RNA-seq datasets of NGFR-overexpressing human melanoma cells, which were reanalyzed in this study, were generated and described in (*23*). Briefly, total RNA of three experimental replicates per condition was isolated using the RNAeasy Kit (Qiagen) and ribonuclease-free deoxyribonuclease (DNase) set (Qiagen) as described in the manufacturer’s protocol. Quality control of total RNA was done using the Agilent RNA ScreenTape assay and the Agilent 4200 TapeStation. Poly-A mRNA was enriched using magnetic beads (TruSeq RNA Library Prep Kit v2) followed by cDNA synthesis and library preparation. RNA-seq was performed with Illumina HiSeq4000 at the Functional Genomics Center Zurich, Switzerland. RNA counts were quantified from single-end reads using STAR aligner, and bioinformatic analysis was performed with EdgeR. Genes were filtered according to thresholds for log_2_[fold change (fc)] ≥ +0.27 and ≤ −0.27 and false discovery rate (FDR) < 0.05. GSEA was performed using the Kyoto Encyclopedia of Genes and Genomes (KEGG) pathway database using the online available WebGestalt software (*80*). Data file S1 contains the list of differentially expressed genes used for GSEA analysis in Fig. 1 (A and B). Enriched pathways and corresponding genes are listed in data file S2. For the analysis of immune-related genes, differentially expressed genes from melanoma cells overexpressing NGFR versus control were intersected with a list containing 844 genes described as directly involved in immunological processes (CD molecules, chemokines, chemokine receptors, and signaling molecules involved in immunity-related cascades) but excluding proteins ubiquitously expressed in nearly all cells, and genes coding for segments of immunoglobulins, as well as B and T cell receptors and MHCs. The gene list was retrieved from the innateDB website [www.innatedb.com (*35*)].

### Human melanoma xenografts

Cultured M010817 human melanoma cells were detached using PBS containing 2 mM EDTA. A total of 3 × 10^5^ cells were resuspended in 100 μl of RPMI 1640 medium and mixed 1:1 with Matrigel matrix (BD Biosciences). In total, 200 μl of the tumor suspension was subcutaneously injected into both flanks of *Foxn1^nu^* nude mice. Seventeen days after engraftment, mice were treated with Dox (2 mg/ml; Sigma-Aldrich) in drinking water plus sucrose (5%) for 6 days to induce NGFR expression in vivo. Tumor volume was measured using a caliper and calculated using the formula: *V* = 2/3 × π × [(*a* + *b*)/4]3 with *a* (millimeters) representing the length and *b* (millimeter) the width of the tumor.

### Flow cytometric analysis of tumor xenografts

Upon sacrifice, tumors were cut into small pieces and dissociated using PBS containing collagenase D (2 mg/ml; Roche) and DNase I (100 mg/ml; Roche) for 2 hours at 37°C. Single cells were separated from remaining tissue using a 40-μm cell strainer (Sigma-Aldrich). Single cells were pelleted and resuspended in fluorescence-activated cell sorting (FACS) buffer [PBS, 2 mM EDTA, and 2% FBS]. To block unspecific binding of antibodies, cells were incubated with anti-CD16/32 blocking antibody (BioLegend) in FACS buffer for 15 min on 4°C. Dead cells were excluded using the LIVE/DEAD Fixable Near-IR Dead Cell Stain Kit (Thermo Fisher Scientific). For immune cell labeling, single-cell suspensions were incubated with fluorophore-conjugated antibodies in Brilliant Stain Buffer (BD Biosciences) for 30 min at 4°C. Cell were washed and fixed with 1.5% paraformaldehyde (PFA). Sample acquisition and compensation was performed on a BD FACSymphony A5 flow cytometer.

### Western blotting

For total protein isolation, cultured cells were harvested by centrifugation and lysed in radioimmunoprecipitation assay buffer (Thermo Fisher Scientific) containing Halt Protease and Phosphatase Inhibitor Cocktail (Thermo Fisher Scientific). Sample homogenization was achieved by using a SONOPULS HD 2070 Ultrasonic Homogenizer (Bandelin). Protein concentrations were determined using the Pierce BCA Protein Assay Kit (Thermo Fisher Scientific) and quantified at a DTX 880 Multimode Detector at 562 nm. Protein samples were supplemented with 4× Laemmli Sample Buffer (Bio-Rad) containing 10% 2-mercaptoethanol and denaturated at 85°C for 5 min. Ten to 20 μg of protein per sample was loaded onto Mini-PROTEAN TGX Precast Gels (Bio-Rad), separated by SDS–polyacrylamide gel electrophoresis and transferred onto nitrocellulose membranes (Bio-Rad). Membranes were probed with primary antibodies in Odyssey blocking buffer (LI-COR Biosciences) overnight at 4°C. Membranes were washed with PBS containing 0.05% Tween 20 (Sigma-Aldrich). Primary antibodies were visualized using secondary antibodies in Odyssey blocking buffer for 45 min at room temperature. Immunoblots were scanned with an Odyssey imaging system (LI-COR Biosciences).

### Flow cytometric analysis of cultured cells

All antibodies are listed in table S1. Dead cells were excluded using Fixable Viability dyes [Aqua (BioLegend) and Red and Near-IR (Thermo Fisher Scientific)]. For surface stainings, cells were incubated with fluorophore-conjugated antibodies in FACS buffer (PBS, 2 mM EDTA, and 2% FBS) or Super Bright Complete Staining Buffer (Thermo Fisher Scientific) for 30 min at 4°C. Thereafter, cells were washed and fixed with 1.5% PFA or BD Cytofix Fixation Buffer (BD Biosciences). For intracellular stainings, cells were fixed and permeabilized with the BD Cytofix/Cytoperm Kit (BD Biosciences) and subsequently incubated with fluorophore-conjugated antibodies for 30 min at 4°C. For compensation, UltraComp eBeads compensation beads or cellular controls were used. Acquisition and compensation were performed on BD FACS Canto II, BD LSR II Fortessa, and BD FACSymphony A5 flow cytometers. Data were exported and analyzed using FlowJo software (version 10, TreeStar Inc.).

### Isolation of human NK cells and in vitro activation

Human NK cells were isolated from buffy coats or whole blood. In a first step, peripheral blood mononuclear cells were obtained by density centrifugation using Histopaque-1077 (Sigma-Aldrich). In a second step, human NK cells were purified using CD56 MicroBeads (Miltenyi) and LS columns (Miltenyi) in a MidiMACS Separator (Miltenyi) according to the manufacturer’s instructions. Purified NK cells were cryopreserved in RPMI 1640 containing 10% dimethyl sulfoxide (DMSO) and 20% heat-inactivated FBS and stored at −140°C. Before experiments, NK cells were thawed and cultured in complete RPMI 1640 supplemented with recombinant human IL-2 (1000 U/ml; Thermo Fisher Scientific) and recombinant human IL-15 (10 ng/ml; Thermo Fisher Scientific) in a humidified incubator at 37°C and 5% CO_2_ for 48 hours.

### HLA haplotyping

DNA was isolated using QIAGEN kits according to the manufacturer’s protocol. HLA typing was performed by a polymerase chain reaction sequence–specific oligonucleotide reverse assay using commercial HLA kits (Fujirebio Diagnostics Inc.) and LIPA Interpretation Software (Fujirebio Diagnostics Inc.).

### In vitro degranulation assays

For degranulation assays, preactivated human NK cells were cocultured with human melanoma cells at an effector to target cell ratio of 1:1 in complete RPMI 1640 containing PE-labeled anti-human CD107a antibody (H4A3, BioLegend) in a humidified incubator at 37°C and 5% CO_2_. For KIR-matched assays, culture medium was supplemented with the metalloproteinase inhibitor TAPI-1 (5 μg/ml; Tocris) to prevent shedding of the CD16 molecule following NK cell activation. Brefeldin A (5 μg/ml; Sigma-Aldrich) and monensin (2 μM; Thermo Fisher Scientific) were added after 1 hour of incubation. After a total of 5 hours of incubation, cells were washed, stained, and analyzed on a BD FACSymphony A5 flow cytometer. UMAP algorithm (*81*) visualization was performed using FlowJo software (version 10, TreeStar Inc.). Data were pregated on live, CD45^+^ cell populations, downsampled to 27,000 cells per condition using the DownSample (v3.3) plugin, and subsequently concatenated per donor. The UMAP plugin (v3.1) for FlowJo was used with the following parameters for dimensionality reduction: “Euclidean,” nearest neighbors: 50; minimum distance: 0.25; number of components: 2.

### In vitro cytotoxic assays

In vitro cytotoxic assays were performed with preactivated human NK cells and human melanoma cells. Before coculture, target cells were labeled with CellTrace Violet (1:2000; BioLegend) for 20 min at 37°C, according to the manufacturer’s instructions. Labeled target cells were cultured together with NK cells at effector to target cell ratios of 8:1, 4:1, and 2:1 for 4 hours in complete RPMI 1640 in a humidified incubator at 37°C and 5% CO_2_. Culture of target cells alone served as negative control. Each experimental condition was performed in duplicates or triplicates. Following coculture, cells were washed, stained, and analyzed on a BD LSR II Fortessa or BD FACSymphony A5 flow cytometer. Effector cells were identified as CD56^+^ or CD45^+^, and target cells were identified as Violet^+^. Dead cells were discriminated from live cells using fixable viability dyes [Red and Near-IR (Thermo Fisher Scientific)]. Specific lysis was calculated by subtracting the frequency of dead targets cells cultured alone (negative control) from the frequency of dead target cells from coculture with effector cells.

### Construction of inducible CRISPR-Cas9 NGFR gene knockout lentivector

Single-guide RNA (sgRNA) sequences used for the generation of Dox-inducible CRISPR-Cas9 lentivector systems are listed in table S1. sgRNA-targeting human NGFR was designed using the Vienna Bioactivity CRISPR score (*82*). A nontargeting sgRNA was used as a control (*83*). sgRNA oligonucleotides were cloned into the all-in-one Dox-inducible Cas9 (iCas9) LentiCRISPR v2 vector (TLCV2, Addgene no. 87360), which was a gift from A. Karpf (*84*, *85*). One Shot Stbl3 Chemically Competent *Escherichia coli* (Thermo Fisher Scientific) were transformed with plenti-iCas9-sgNGFR or plenti-iCas9-sgCtr plasmid. Plasmid isolation was performed using the NucleoBond Xtra Midi Plus EF Kit (Macherey-Nagel). Correct sequence insertion was confirmed by Sanger sequencing (Microsynth, Switzerland).

### Lentivirus production and melanoma cell infection

For lentivirus production, HEK-293T cells were transfected with plenti-iCas9 plasmid (1 μg/ml) in combination with psPAX2 packaging plasmid (0.55 μg/ml; Addgene no. 12260) and pMD2.G envelope–expressing plasmid (0.25 μg/ml; Addgene no. 12259) using calcium phosphate precipitation. Supernatants containing viral particles were harvested at 24 and 48 hours after transfection, filtered through 0.45-μm filters, and added to target cells 1:1 in complete RPMI 1640 supplemented with polybrene (8 μg/ml; H9268, Sigma-Aldrich). After 24 hours, medium was replaced, and transduced cells were selected in puromycin (1.5 μg/ml) for 10 days and further maintained at puromycin (0.5 μg/ml). Gene knockouts were induced by treatment of cells with Dox (1 μg/ml) in complete RPMI 1640 for 6 days.

### In vivo cytotoxic assay

Melanoma cells were pretreated in vitro and labeled as indicated. Violet^dim^ cell populations were stained with 0.5 μM CellTrace Violet (Thermo Fisher Scientific), whereas Violet^bright^ cell populations were labeled with 5 μM CellTrace Violet (Violet^bright^) for 20 min according to the manufacturer’s instructions. Violet^bright^ cells were mixed with equal numbers of Violet^dim^ cells. The cell suspension was then either mixed with equal numbers of preactivated human NK cells or an equal volume of PBS as vehicle control. Cell suspensions were kept on ice for maximal 30 min until 3 to 4 × 10^6^ tumor cells (1.5 to 2 × 10^6^ cells per population) mixed with 3 to 4 × 10^6^ human NK cells or PBS were intraperitoneally injected into NSG mice. Eight hours after inoculation, cells were harvested from recipient mice by peritoneal lavage. Harvested cells were pelleted, washed, and labeled for murine CD45 (BUV395 Rat Anti-Mouse CD45, BD Biosciences) and human CD45 (PE anti-human CD45 Antibody, BioLegend) for 30 min at 4°C. Dead cells were excluded using the LIVE/DEAD Fixable Near-IR Dead Cell Stain Kit (Thermo Fisher Scientific). Cell suspensions were fixed using Cytofix (BD Biosciences). Sample acquisition was performed on a BD FACSymphony A5 cytometer (BD Biosciences). The percent specific lysis was determined by loss of the Violet^dim^ population compared to the Violet^bright^ population using the formula: {1 − [(%Violet^bright^/%Violet^dim^) in PBS mice]/[(%Violet^bright^/%Violet^dim^) in NK mice]} × 100.

### LC-MS–based lipidomic analysis

Liquid chromatography–mass spectrometry (LC-MS)–based lipidomic analysis was performed with M010817 CMVTOEV and CMVTONGFR cells pretreated with Dox (1 μg/ml) for 24 hours. Cells were detached with PBS 2 mM EDTA, washed, and resuspended in 1-butanol/methanol (1:1). Cells were vortexed for 15 s and subsequently sonicated on ice with a pulse of 3× 10 s and 1× 30 s at an amplitude of 15% using a SONOPULS HD 2070 Ultrasonic Homogenizer (Bandelin). Cell extracts were centrifuged at 16,000*g*, 20°C for 10 min to remove macromolecules and subsequently diluted (1:3) with water/methanol (1:2) solution. The dilution was vortexed and centrifuged (16,000*g*, 20°C, 10 min). One hundred microliters of the supernatant was transferred to a glass vial with narrowed bottom (Total Recovery Vials, Waters) for LC-MS injection.

Lipids were separated on a nanoAcquity UPLC (Waters) equipped with a HSS T3 capillary column (150 μm by 30 mm, 1.8-μm particle size; Waters), applying a gradient of 5 mM ammonium acetate in water/acetonitrile 95:5 (A) and 5 mM ammonium acetate in isopropanol/acetonitrile 90:10 (B) from 5 to 100% B over 10 min. The following 5-min conditions were kept at 100% B, followed by 5-min reequilibration to 5% B. The injection volume was 1 μl. The flow rate was constant at 2.5 μl/min. The UPLC was coupled to QExactive mass spectrometer (Thermo Fisher Scientific) by a nanoESI source. MS data were acquired using positive polarization and data-dependent acquisition. Full scan MS spectra were acquired in profile mode from 80 to 1200 mass/charge ratio with an automatic gain control target of 1 × 10^6^, an Orbitrap resolution of 70,000, and a maximum injection time of 200 ms. The five most intense charged (*z* = +1 or +2) precursor ions from each full scan were selected for collision induced dissociation fragmentation. Precursor was accumulated with an isolation window of 0.4 Da, an automatic gain control value of 5 × 10^4^, a resolution of 17,500, and a maximum injection time of 50 ms and fragmented with a normalized collision energy of 20, 30, and 40 (arbitrary unit). Generated fragment ions were scanned in the linear trap. Minimal signal intensity for MS2 selection was set to 500. Datasets were evaluated with Progenesis QI software (Nonlinear Dynamics), which aligns the ion intensity maps based on a reference dataset, followed by a peak picking on an aggregated ion intensity map. Detected ions were identified on the basis of accurate mass, detected adduct patterns, and isotope patterns by comparing with entries in the LipidMaps Data Base (LM) and KEGG database. Considered adducts were M + H, M + NH_4_, 2 M + H, and M + H-H_2_O. A mass accuracy tolerance of 5 parts per million was set for the searches. Fragmentation patterns were considered for the identifications of metabolites. Putative identifications were further ranked on the basis of mass error (observed mass − exact mass), isotope similarity (observed versus theoretical).

Volcano plots were generated by using MetaboAnalyst 5.0 (www.metaboanalyst.ca). Significantly changed metabolites were identified by fc > 1.5 and *P* < 0.05.

### Lipid metabolism inhibitors

For in vitro and in vivo cytotoxic assays with lipid metabolism inhibitors, melanoma cells were treated with 1 μM Lovastatin (Abcam) or 10 μM MF438 (Merck Millipore) in combination with Dox (1 μg/ml) for the indicated time points.

### RNA interference

M010817 cells were transfected using the JetPRIME transfection kit (Polyplus) according to the manufacturer’s guidelines. A375mel cells were transfected by electroporation using the NEON transfection system (Thermo Fisher Scientific). Final concentrations of 20 nM siRNA were used to transfect cells at 50% confluency. siRNAs used for experiments are listed in table S1. Twenty-four hours after transfection, culture medium was replaced with fresh, complete RPMI 1640 containing Dox (1 μg/ml), and cells were incubated for another 48 hours before gene knockdown was validated by Western blot.

### Laurdan probe and image analysis

M010817 cells were seeded on poly-l-lysine (R&D Systems)–coated cover slides and cultured in complete RPMI containing Dox (1 μg/ml) for 48 hours. Thereafter, cells were incubated with 50 μM Laurdan probe (Sigma-Aldrich) in serum-free DMEM for 1 hour in a humidified incubator at 37°C and 5% CO_2_. Following incubation, cells were washed and fixed with 4% PFA and imaged with a 20× objective at a LEICA SP8 MP Dive Falcon microscope. Laurdan probe was excited at 800 nm, and emission was measured using two detection channels: detection channel 1 at 410 to 460 nm and detection channel 2 at 480 to 530 nm. Image analysis was performed using the Fiji software. To quantify lipid packing, same sized regions of interests were drawn at the cell membranes. Intensities of the separated detection channels were measured, and GP values were calculated according to the formula shown in fig. S6B. Image analysis was performed in a blinded manner.

### Metastasis assay with adoptively transferred human NK cells

For experimental metastasis assays, A375mel EV and NGFR melanoma cells were pretreated in vitro with Dox (1 μg/ml) for 48 hours in complete RMPI 1640. NSG mice were tail vein–injected with 2 × 10^6^ A375mel cells alone or together with 3 × 10^6^ preactivated human NK cells in a total volume of 100 of μl PBS using a 29-gauge 1/2 needle. To enhance survival of adoptively transferred human NK cells, NSG mice were intraperitoneally injected with 2.5 μg of recombinant human IL-15 (Miltenyi Biotec) twice per week. Seven days after intravenous injections, mice were euthanized with CO_2_, and lungs and blood samples were collected. Lungs were cut into small pieces, and lung tissue was dissociated using Liberase (0.25 mg/ml; Roche) RPMI 1640 for 40 min at 37°C, followed by the addition of DNase I (0.2 mg/ml; Roche) for 15 min at 37°C. Single cells were separated from remaining tissue using a 40-μm cell strainer (Sigma-Aldrich). Single cells were washed and fixed with BD Cytofix Fixation Buffer (BD Biosciences). Following fixation, cells were pelleted and resuspended in 2 ml of FACS buffer (PBS, 2 mM EDTA, and 2% FBS). Before acquisition, exact numbers of CountBright Absolute Counting Beads (Thermo Fisher Scientific) were added to the samples. Samples were analyzed for GFP-positive cells on a BD FACS Canto II. Blood samples were stained, fixed, and acquired on a BD FACSymphony A5 flow cytometer.

### Statistical analysis

Statistical analyses were conducted using GraphPad Prism 8. All quantitative analyses comparing two groups were performed by unpaired, two-tailed Student’s *t* tests. For all statistical analyses, the expected variance was similar between the groups that were compared, and significance was accepted at the 95% confidence level (∗*P* < 0.05, ∗∗*P* < 0.01, and ∗∗∗*P* < 0.001). Unless otherwise indicated, data are presented as means ± SD.

## Notes

View/request a protocol for this paper from *Bio-protocol*.
