# Supplementary material for: Escape from NK cell tumor surveillance by NGFR-induced lipid remodeling in melanoma
Source: Sci Adv. 2023 Jan 13;9(2):eadc8825. doi: 10.1126/sciadv.adc8825 (PMC9839334; doi:10.1126/sciadv.adc8825)
Supplement: Supplementary file 1 — Figs. S1 to S6 Table S1 [file sciadv.adc8825_sm.pdf]

Science Advances
AAAS

Supplementary Materials for

Escape from NK cell tumor surveillance by NGFR-induced lipid remodeling in melanoma

Julia Lehmann *et al.*

Corresponding author: Johanna Diener, johanna.diener@anatomy.uzh.ch;
Lukas Sommer, lukas.sommer@anatomy.uzh.ch

Sci. Adv. **9**, eadc8825 (2023)
DOI: 10.1126/sciadv.adc8825

The PDF file includes:

Figs. S1 to S6
Table S1
Legends for data files S1 to S3

Other Supplementary Material for this manuscript includes the following:

Data files S1 to S3

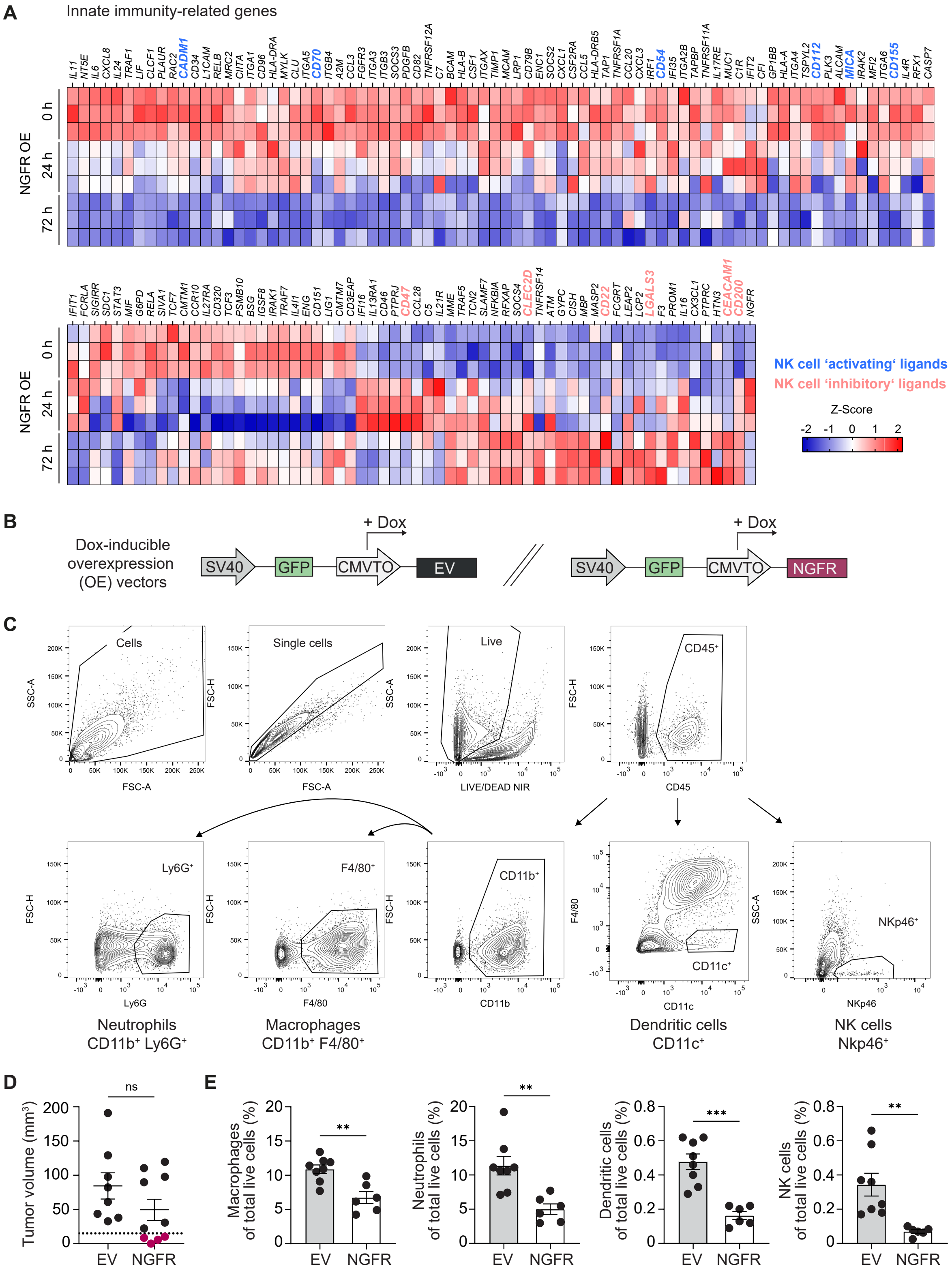

Fig. S1. *(Legend on next page)*

Fig. S1. Analysis of immune cell infiltration in tumor xenografts.

(**A**) Heatmap showing expression of innate immunity-related genes from Figure 1C after 0 h, 24 h and 72 h of NGFR overexpression in M010817 cells. Genes encoding putative NK cell activating and inhibitory ligands are highlighted in blue and red, respectively. Shown are the Z-Scores for n = 3. (**B**) Schematic illustration of the lentiviral NGFR-overexpression vector (NGFR) and the control empty vector (EV) used to engineer the patient-derived melanoma cell line M010817. NGFR and EV cassettes are under the control of a doxycycline (Dox)-inducible CMVTetOperon (CMVTO) promoter. The GFP reporter is constitutively expressed by the SV40 promoter. (**C**) Flow cytometry gating strategy for discriminating different immune cell subtypes. Macrophages were defined by expression of $CD11b^{+}$ $F4/80^{+}$, neutrophils by $CD11b^{+}$ $Ly6G^{+}$, dendritic cells by $CD11c^{+}$ and NK cells by expression of $NKp46^{+}$. (**D**) Volumes of all xenograft tumors. Tumors, which were excluded from the analysis, are shown in red. Mean ± SEM. (**E**) Frequencies of infiltrating immune cells of total live cells in tumors. Mean ± SEM. P-values were calculated by unpaired, two-tailed Student's t-test with $*p < 0.05$, $**p < 0.01$, $***p < 0.001$.

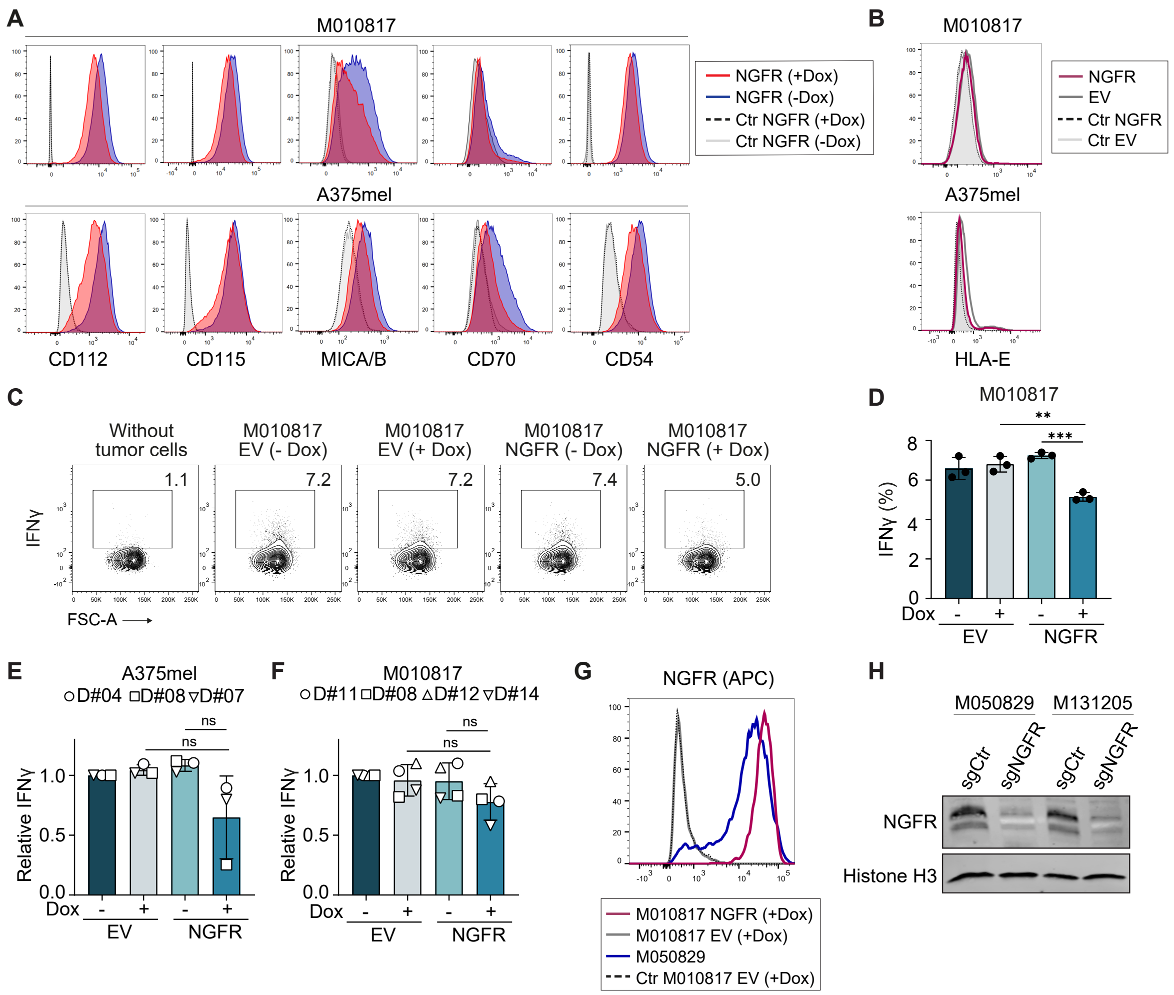

Fig. S2. NGFR deregulates NK cell ligands and reduces NK cell activity.

(**A**) Representative histograms showing fluorescent intensities of ligands for activating NK cell receptors on NGFR-induced (red, +Dox) and non-induced (blue, -Dox) melanoma cells 72 h after Dox treatment. (**B**) Representative histograms displaying fluorescent intensities of HLA-E on NGFR (red) and EV (grey) melanoma cells 72 h after Dox induction. (**C**) Representative flow cytometry analysis of intracellular IFNγ in human NK cells (D#11) after 5 h of coculture with vector-engineered M010817 cells (after 72 h Dox induction). (**D**) Quantification of (C) showing technical triplicates. Mean ± SD. (**E** and **F**) Summary of relative IFNγ expression in human NK cells derived from four different donors after coculture with A375mel (E) and M010817 (F). Data are normalized to expression levels of NK cells cocultured with EV control cells (- Dox). Circle, square and triangle symbols represent mean values of technical duplicates or triplicates using the indicated NK cell donors (D#). N = 3 - 4, Mean ± SD. (**G**) Flow cytometry analysis comparing NGFR levels on untreated M050829, M010817 EV and M010817 NGFR cells 72 h after Dox induction. Unstained M010817 EV cells (+ Dox 72h) served as control (Ctr). (**H**) Western blot analysis of NGFR in M050829 and M131205 six days after CRISPR-induced NGFR gene knockout (Dox-inducible single guide control (sgCtr) or single guide targeting NGFR (sgNGFR)). P-values were calculated by unpaired, two-tailed Student's t-test with $*p < 0.05$, $**p < 0.01$, $***p < 0.001$.

A

HLA-Haplotyping

Target	HLA-A	HLA-B	HLA-C	Bw	HLA-B -21	C group
A375	01:01, 02:195	44:39, 57:01	16:01, 06:02	Bw4-80T, Bw4-80I	T/T	C1/C2
M01	11:01	55:01	03:67, 14:15	Bw6	T	C1/C1

Effector	HLA-A	HLA-B	HLA-C	Bw	HLA-B -21	C group
Donor A	25:01, 30:01	44:02, 51:01	15:24, 12:03	Bw4-80T, Bw4-80I	T/T	C1/C2
Donor B	03:01, 29:02	44:03, 56:01	01:02, 16:01	Bw4-80T, Bw6	T/T	C1/C1

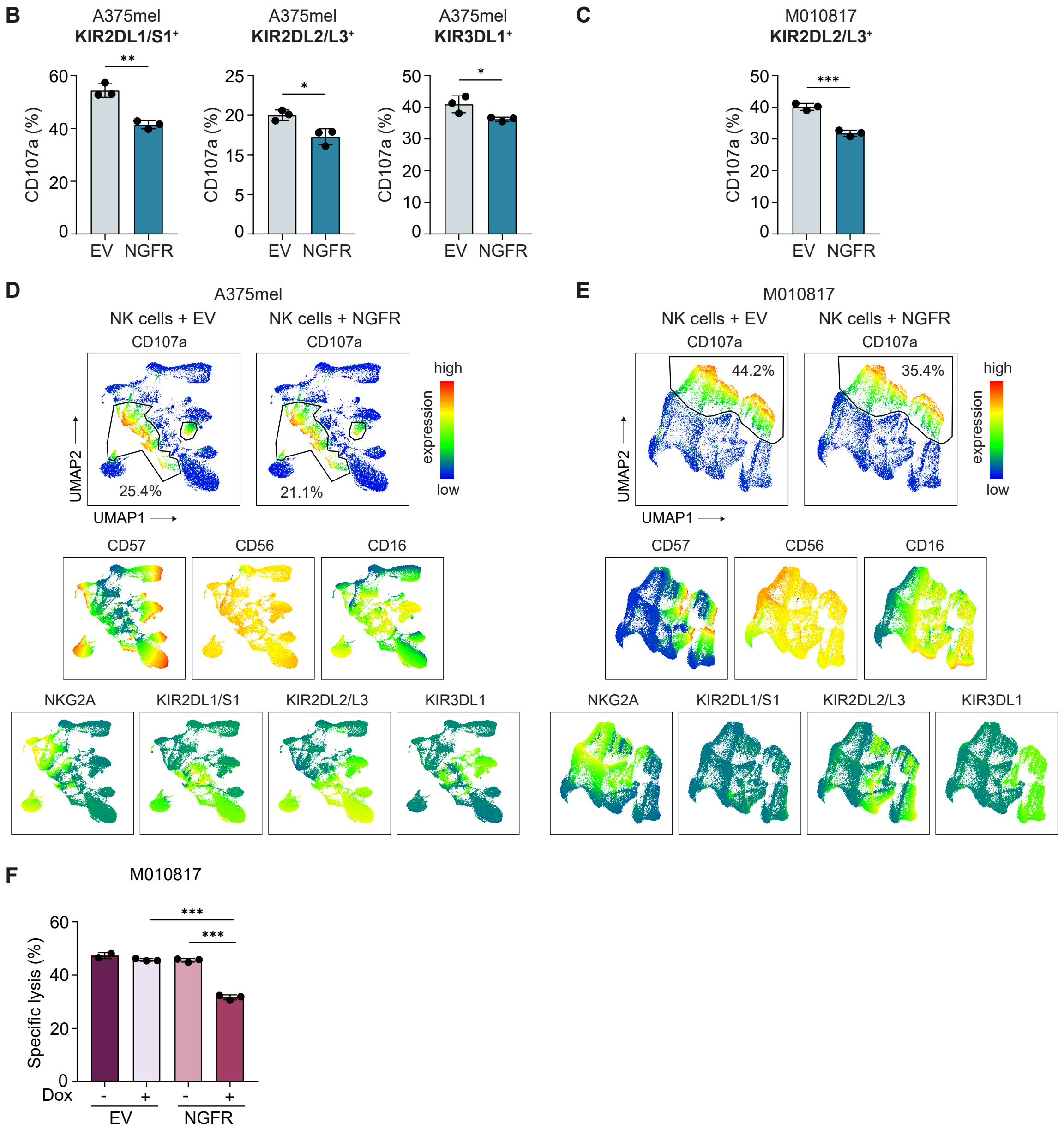

Fig. S3. *(Legend on next page)*

Fig. S3. Activation of KIR-matched NK cells.

(**A**) Table displaying HLA-haplotypes of melanoma cells (A375: A375mel, M01: M010817) and NK cell Donor A and B. (**B**) Frequency of CD107a on indicated KIR-positive Donor A-derived NK cells after 5 h of coculture with A375mel cells. Shown are technical n = 3, Mean ± SD. (**C**) Frequency of CD107a on indicated KIR-positive Donor B-derived NK cells after 5 h of coculture with M010817 cells. Shown are technical n = 3, Mean ± SD. (**D**) UMAP visualization of single, live, human $CD45^+$ Donor A-derived NK cells after coculture with A375mel cells. Upper panel depicts relative CD107a expression after coculture with indicated target cells. Lower panels display relative marker expression. (**E**) Same as in (D) for Donor B-derived NK cells after coculture with M010817 cells. (**F**) Specific lysis of M010817 tumor cells after coculture with KIR-matched Donor B-derived NK cells at a 4:1 effector: target ratio. Data are representative of three independent experiments. Mean ± SD. P-values were calculated by unpaired, two-tailed Student's t-test with $*p < 0.05$, $**p < 0.01$, $***p < 0.001$.

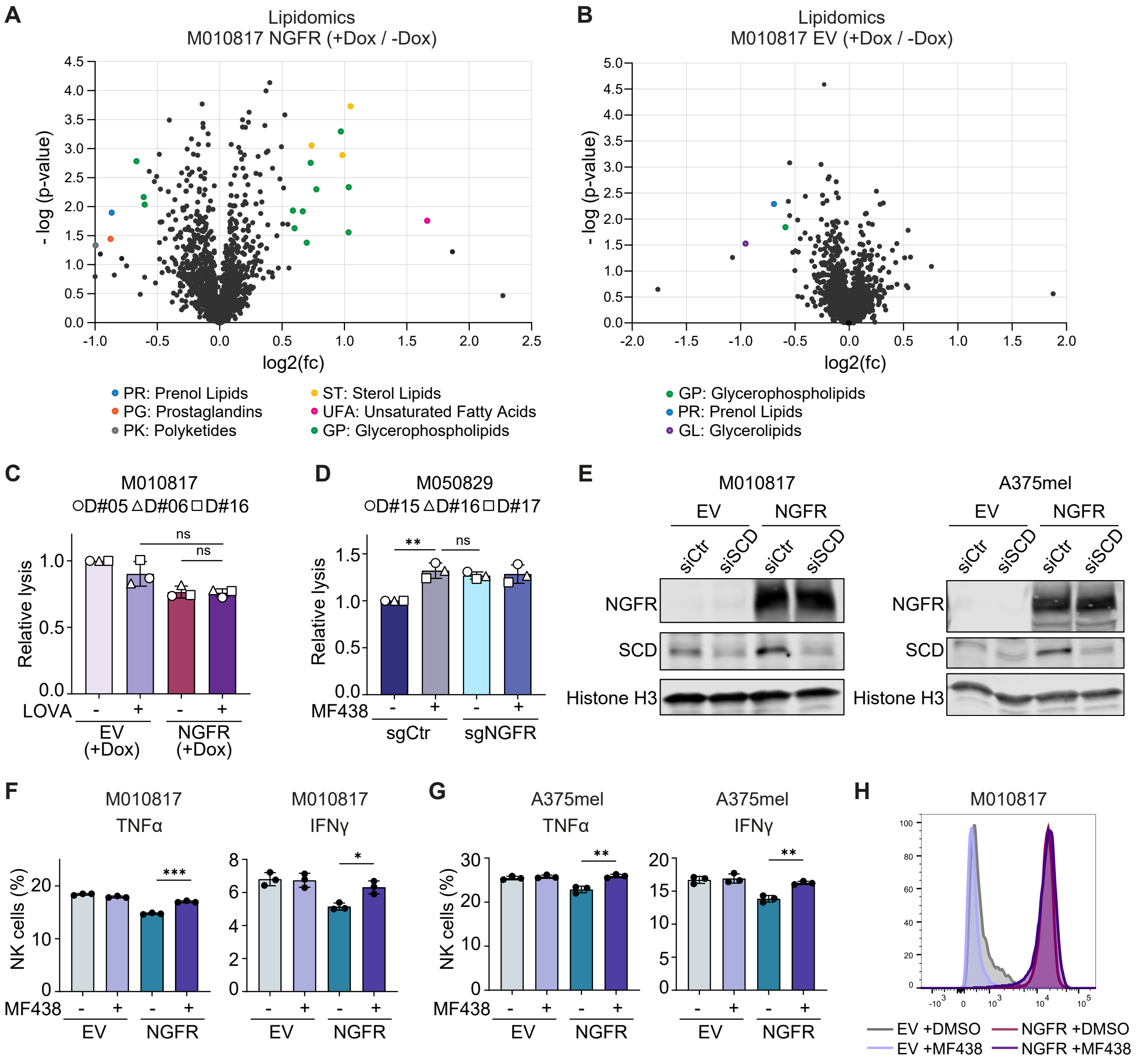

Fig. S4. NGFR alters lipid composition of melanoma cells.

(**A** and **B**) Volcano plot of LC/MS-based untargeted lipidomic data of M010817 cells (A) overexpressing NGFR (24 h Dox treatment over vehicle) or (B) EV control (24 h Dox treatment over vehicle) to rule out a Dox effect on lipids. Highlighted are significantly changed metabolites and the corresponding lipid classes are annotated below. Thresholds were set for p value > 0.05 and fc > 1.5. (**C**) Relative lysis of vector-engineered M010817 simultaneously pretreated with Dox and 1 μM lovastatin (LOVA) or vehicle (DMSO) for 48 h. (**D**) Relative lysis of M050829 sgCtr and sgNGFR cells treated with Dox for 6 days and 10 μM MF438 or vehicle (DMSO) for the last 48 h. (C and D) Shown are the mean values of three independent experiments (with technical n = 3) using indicated NK cell donors at 4:1 effector: target cell ratios. Mean ± SD. (**E**) Western blot analysis to verify upregulation of SCD upon NGFR overexpression and subsequent knock down efficiency after siSCD application (for 72 h) in M010817 and A375mel EV and NGFR cells (treated with Dox for 48 h). (**F** and **G**) Flow cytometry analysis of intracellular TNFα and IFNγ on NK cells after coculture with M010817 (F) and A375mel (G) simultaneously pretreated with 10 μM MF438 or vehicle (DMSO) and Dox for 48 h. Technical n = 3, Mean ± SD. (**H**) Flow cytometry analysis showing NGFR levels on M010817 cells treated with MF438 or DMSO and Dox for 48 h. P-values were calculated by unpaired, two-tailed Student's t-test with ∗p < 0.05, ∗∗p < 0.01, ∗∗∗p < 0.001.

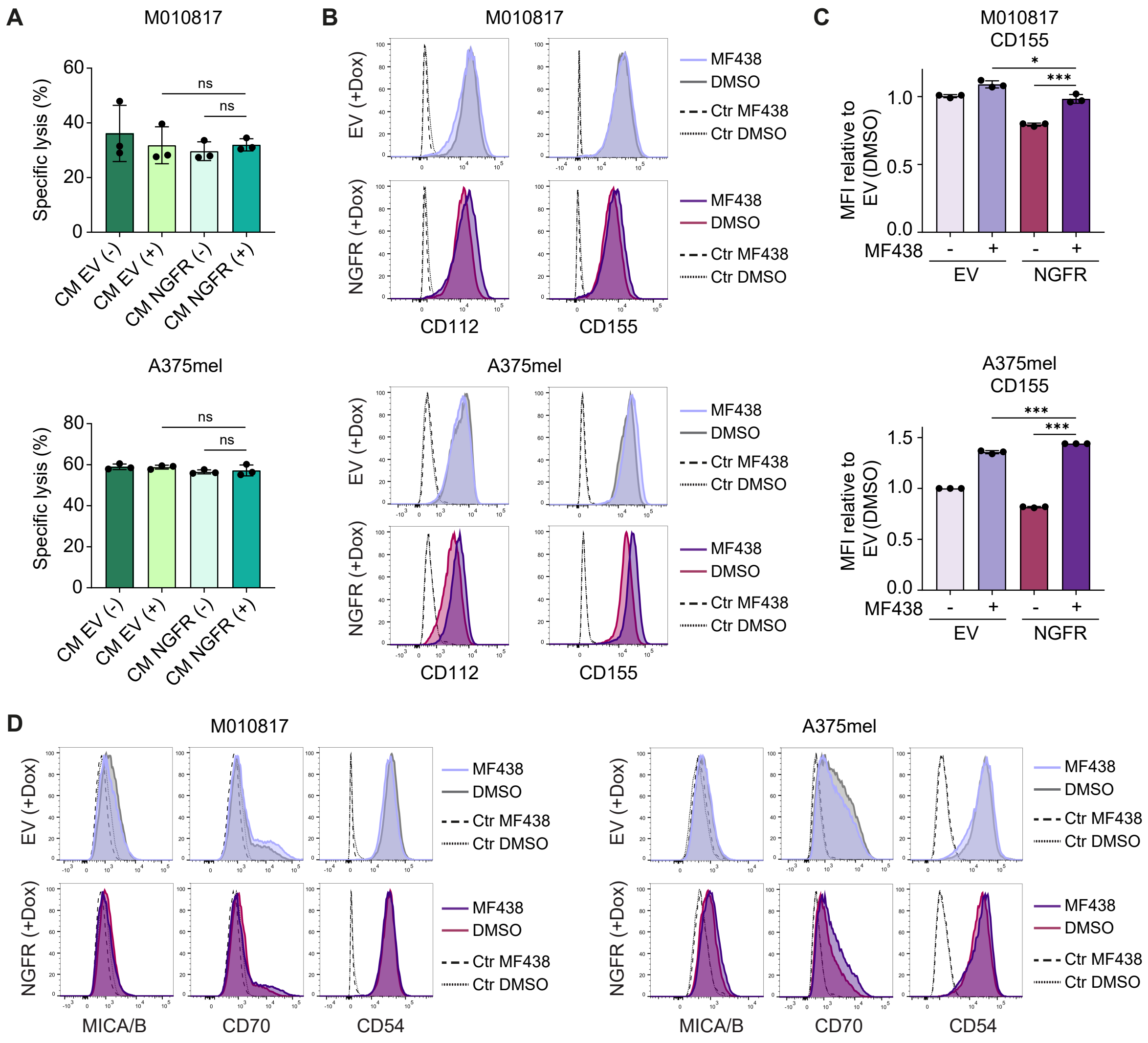

Fig. S5. SCD inhibition upregulates CD112 and CD155 on NGFR-overexpressing cells.

(**A**) Specific lysis of 'wild-type' (non-engineered) M010817 and A375mel cells after 4 h of co-culture with human NK cells in presence of conditioned media (CM) from Dox-treated ('+') or vehicle-treated ('-') (72 h) vector-engineered (EV or NGFR) M010817 or A375mel cells, respectively. Effector: target cell ratio 4:1. Technical n = 3, Mean ± SD. (**B**) Fluorescent intensity of CD112 and CD155 on EV control cells and NGFR OE cells treated with MF438 or DMSO (plus Dox for 48 h). Unstained cells served as control (Ctr). (**C**) Quantification of relative MFI of CD155 on M010817 and A375mel cells treated with MF438 or vehicle (DMSO) in combination with Dox for 48 h. Technical n = 3, Mean ± SD.(**D**) Fluorescent intensity of ligands for activating NK cell receptors on NGFR OE and EV cells treated with MF438 or DMSO (plus Dox for 48 h). Unstained cells served as control (Ctr). P-values were calculated by unpaired, two-tailed Student's t-test with $*p < 0.05$, $**p < 0.01$, $***p < 0.001$.

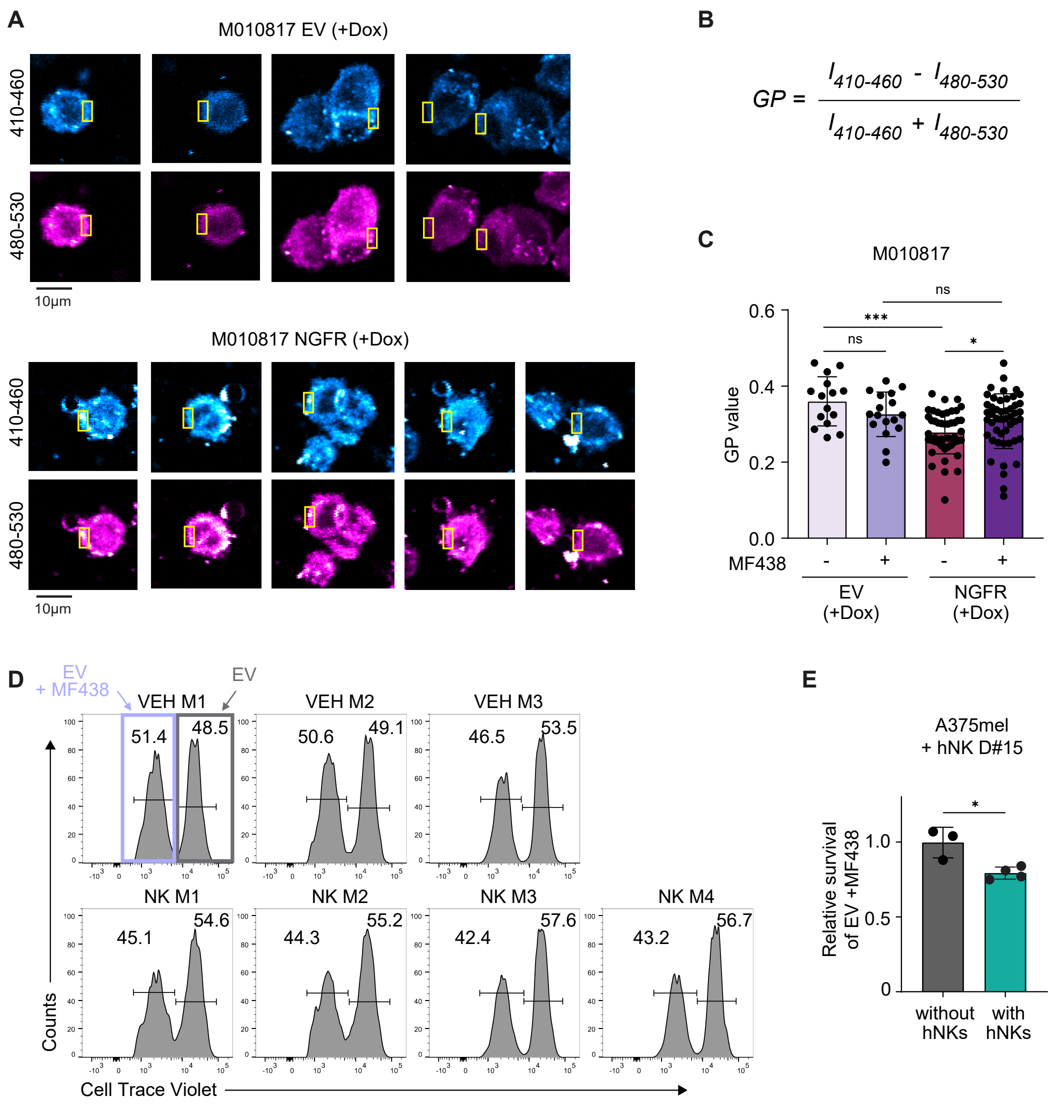

Fig. S6. NGFR alters membrane polarization of melanoma cells.

(**A**) Representative images of Laurdan dye emission at 410-460 nm and emission at 480-530 nm for EV and NGFR OE M010817 cells (treated with Dox for 48 h). Emission intensities of plasma membranes were measured within the yellow squares and generalized polarization (GP) was quantified using the formula in (**B**). (**C**) Quantification of GP values from EV and NGFR OE M010817 cells simultaneously pretreated with Dox and 10 µM MF438 or vehicle (DMSO) for 48 h. (**D**) Flow cytometry of Cell Trace Violet-labeled A375mel cells at recovery 8 h after intraperitoneal injection into NSG mice. Prior injection, A375mel EV cells pretreated with Dox and MF348 or DMSO for 48 h were labeled with different concentrations of Cell Trace Violet (EV +MF438 = Violet^{dim}, EV +DMSO = Violet^{bright}). Labeled cell populations were mixed and injected together with human NK cells or PBS as vehicle control into the peritoneum of NSG mice (1:1 ratio of effector: target cell mix). (**E**) Graph displays relative survival of EV cells +MF438 over EV +DMSO in mice with human NK cells normalized to mice without human NK cells. Mean ± SD. P-values were calculated by unpaired, two-tailed Student's t-test with $*p < 0.05$, $**p < 0.01$, $***p < 0.001$.

Table S1.

Resources	Source	Catalogue Number
**Antibodies**		
Anti-mouse CD16/32 TruStain FcX™ (93)	BioLegend	Cat#101319
Anti-mouse CD11b BUV737 (M1/70)	BD Biosciences	Cat#612801
Anti-mouse CD11c APC (N418)	BioLegend	Cat#117309
Anti-mouse CD45 BUV395 (30-F11)	BD Biosciences	Cat#564279
Anti-mouse CD335/NKp46 BV421 (29A1.4)	BD Biosciences	Cat#652850
Anti-mouse F4/80 PE (BM8)	BioLegend	Cat#123109
Anti-mouse Ly-6G PE-Cy7 (1A8)	BioLegend	Cat#127618
Anti-human CD112 PE (TX31)	BioLegend	Cat#337409
Anti-human CD155 PE (SKII.4)	BioLegend	Cat#337609
Anti-human MICA/MICB APC (6D4)	BioLegend	Cat#320907
Anti-human MICA/MICB PE (6D4)	BioLegend	Cat#320906
Anti-human CD70 APC (113-16)	BioLegend	Cat#355109
Anti-human CD70 PE (113-16)	BioLegend	Cat#355103
Anti-human CD54 PB (HCD54)	BioLegend	Cat#322715
Anti-human CD54 PE (HCD54)	BioLegend	Cat#322707
Anti-human HLA-A,B,C APC-Cy7 (W6/32)	BioLegend	Cat#311425
Anti-human HLA-A,B,C PE (W6/32)	BioLegend	Cat#311405
Anti-human HLA-E PE-Dazzle 594 (3D12)	BioLegend	Cat#342615
Anti-human CD16 BUV737 (3G8)	BD Biosciences	Cat#612787
Anti-Human CD45 BUV395 (HI30)	BD Biosciences	Cat#563791
Anti-human CD45 PE (HI30)	BioLegend	Cat#304008
Anti-human CD56 BUV563 (NCAM16.2)	BD Biosciences	Cat#612929
Anti-human CD56 APC (HCD56)	BioLegend	Cat#318310
Anti-human CD107a PE (H4A3)	BioLegend	Cat#328608
Anti-human CD271 APC (ME20.4-1.H4)	Miltenyi Biotec	Cat#130-113-418
Anti-human IFN-γ BV786 (4S.B3)	BD Biosciences	Cat#563731
Anti-human TNF-α APC-Cy7 (MAb11)	BioLegend	Cat#502944
Anti-human CD158b1/b2,j PE-Cy5.5 (GL183)	Beckman Coulter	Cat#A66900
Anti-human CD158a,h PE-Cy7 (EB6B)	Beckman Coulter	Cat#A66899
Anti-human CD158e1 Alexa Fluor 700 (DX9)	BioLegend	Cat#312712
Anti-human DNAM-1 (DX11)	Abcam	Cat#ab33397
Mouse anti-γ-Tubulin (GTU-88)	Sigma-Aldrich	Cat#T6557
Rabbit anti-p75 NGF receptor (extracellular) (polyclonal)	Alomone Labs	Cat#ANT-007
Mouse anti-Histone H3 (96C10)	Cell Signaling	Cat#3638S
Mouse anti-SCD1 (CD.E10)	Abcam	Cat#ab19862
IRDye 680LT donkey anti-rabbit IgG (polyclonal)	LI-COR Biosciences	Cat#926-68023
IRDye 800CW donkey anti-mouse IgG (polyclonal)	LI-COR Biosciences	Cat#926-32212
**Reagents**		
Human IL-2 recombinant protein	ThermoFisher Scientific	Cat#RP-8608
Human IL-15 recombinant protein	ThermoFisher Scientific	Cat#BMS319
Human IL-15 recombinant protein	Miltenyi Biotec	Cat#130-095-765
TAPI-1 (ADAM-17 (TACE) and MMP inhibitor)	Tocris	Cat#6162
Histopaque®-1077	Sigma-Aldrich	Cat#10771
Monensin	ThermoFisher Scientific	Cat#00-4505-51
Brefeldin A	Sigma-Aldrich	Cat#B5936
Brilliant Stain Buffer	BD Biosciences	Cat#563794
Super Bright Complete Staining Buffer	ThermoFisher Scientific	Cat#SB-4401-42
BD Cytofix/Cytoperm™ Fixation/Permeablization Kit	BD Biosciences	Cat#554714
BD Cytofix™ Fixation Buffer	BD Biosciences	Cat#554655

Matrigel matrix	BD Biosciences	Cat#356234
Collagenase D	Roche	Cat#11088866001
DNase I	Roche	Cat#10104159001
Liberase™ DH Research Grade	Roche	Cat#5401054001
Phosphate Buffered Saline (PBS), pH 7.4	ThermoFisher Scientific	Cat#10010015
RPMI 1640 Medium	ThermoFisher Scientific	Cat#42401018
DMEM	ThermoFisher Scientific	Cat#41965
L-Glutamine	ThermoFisher Scientific	Cat#25030024
Penicillin-Streptomycin	ThermoFisher Scientific	Cat#15140122
Fetal Bovine Serum (FBS)	ThermoFisher Scientific	Cat#16140
Ethylenediaminetetraacetic acid (EDTA)	ThermoFisher Scientific	Cat#AM9261
Doxycycline	Sigma-Aldrich	Cat#D9891
Dimethyl sulfoxide (DMSO)	Sigma-Aldrich	Cat#D8418
Laemmli Sample Buffer, 4x	Bio-Rad	Cat#1610747
Odyssey Blocking Buffer	LI-COR Biosciences	Cat# 927-40000
RIPA Buffer	ThermoFisher Scientific	Cat#89900
Halt™ Protease and Phosphatase Inhibitor Cocktail	ThermoFisher Scientific	Cat#78440
Tris/Glycine Buffer, 10X	Bio-Rad	Cat#1610734
Tris/Glycine/SDS Buffer, 10X	Bio-Rad	Cat#1610732
TWEEN 20	Sigma-Aldrich	Cat#P1379
Polybrene	Santa Cruz Biotechnology	Cat#sc-134220
Puromycin	ThermoFisher Scientific	Cat#A11138-02
MF438	Merck Millipore	Cat#569406
Lovastatin	Abcam	Cat#ab120614
Laurdan probe (6-Dodecanoyl-N,N-dimethyl-2-naphthylamine)	Sigma-Aldrich	Cat#40227
Cultrex Poly-L-Lysine	R&D Systems	Cat#3438-100-01
UltraComp eBeads™ compensation beads	ThermoFisher Scientific	Cat#01-2222-41
CountBright™ Absolute Counting Beads	ThermoFisher Scientific	Cat#C36950
CD56 MicroBeads, human	Miltenyi Biotec	Cat#130-050-401
Pierce™ BCA Protein Assay Kit	ThermoFisher Scientific	Cat#23227
Zombie Aqua™ Fixable Viability Kit	BioLegend	Cat#423101
LIVE/DEAD™ Fixable Red Dead Cell Stain Kit	ThermoFisher Scientific	Cat#L34971
LIVE/DEAD™ Fixable Near-IR Dead Cell Stain Kit	ThermoFisher Scientific	Cat#L34975
CellTrace™ Violet Cell Proliferation Kit	ThermoFisher Scientific	Cat#C34571
JetPRIME Transfection Kit	Polyplus	Cat#114-15
**Biological samples and cell lines**		
Human Buffy Coats	Zurich blood donation services (ZHBDS)	BASEC-NR: Req-2020-00883
Whole Blood Samples	Healthy adult volunteers	BASEC-NR: Req-2020-00883
Human A375mel cell line	ATCC	Cat#CRL-1619; RRID: CVCL_0132
Human HEK-293T cell line	ATCC	Cat#CRL-3216; RRID: CVCL_0063
Human M000921 short-term cell culture	URPP Live Tumor Cell Biobank, UZH	N/A
Human M010817 short-term cell culture	URPP Live Tumor Cell Biobank, UZH	N/A
Human M050829 short-term cell culture	URPP Live Tumor Cell Biobank, UZH	N/A
Human M070203 short-term cell culture	URPP Live Tumor Cell Biobank, UZH	N/A
Human M121224 short-term cell culture	URPP Live Tumor Cell Biobank, UZH	N/A

Human M130429 short-term cell culture	URPP Live Tumor Cell Biobank, UZH	N/A
Human M131205 short-term cell culture	URPP Live Tumor Cell Biobank, UZH	N/A
Human M151213 short-term cell culture	URPP Live Tumor Cell Biobank, UZH	N/A
**Recombinant DNA and oligonucleotides**		
TLCV2	Addgene, ref. *(84)*	Cat#87360
psPAX2	Addgene, D. Trono Lab	Cat#12260
pMD2.G	Addgene, D. Trono Lab	Cat#12259
sgCtr: GTCCACCCTTATCTAGGCTA	Ref. *(83)*	N/A
sgNGFR: AGACCTCATAGCCAGCACGG	This paper	N/A
Silencer™ Select Negative Control No. 1 siRNA	ThermoFisher Scientific	Cat#4390844
siSCD	LabForce AG	Cat#4023-sc-36464
**Softwares**		
FlowJo (v10.6.2)	FlowJo	https://www.flowjo.com
GraphPad Prism (v8.4.3)	GraphPad Software	https://www.graphpad.com:443/
Fiji	Image J	https://fiji.sc/
WebGestalt (2019)	Ref. *(80)*	http://www.webgestalt.org/
MetaboAnalyst (5.0)	MetaboAnalyst	https://www.metaboanalyst.ca/

Data file S1. (separate file) Differentially expressed genes upon NGFR overexpression.

List of differentially expressed genes from RNA-seq of Dox-inducible NGFR-overexpressing M010817 cells 24 h and 72 h after Dox induction (comparing Dox-induced versus non-induced cells). Genes were filtered according to thresholds for Log2(fc) $\geq +0.27$ and ≤ -0.27, FDR < 0.05.

Data file S2. (separate file) Enriched pathways and corresponding genes from the GSEA.

List of enriched pathways and corresponding genes from the GSEA of NGFR-overexpressing M010817 cells (24 h after Dox induction). Analysis is based on the KEGG pathway database.

Data file S3. (separate file) Differentially expressed lipids upon NGFR overexpression.

List of differentially expressed lipids from LC/MS-based lipidomic analysis of Dox-inducible NGFR-overexpressing M010817 cells and EV control M010817 cells 24 h after Dox induction (comparing Dox-induced versus non-induced cells).
